# Interaction of Mesonivirus and Negevirus with arboviruses and the RNAi response in *Culex tarsalis*-derived cells

**DOI:** 10.1186/s13071-023-05985-w

**Published:** 2023-10-13

**Authors:** Eric Agboli, Jonny Schulze, Stephanie Jansen, Daniel Cadar, Vattipally B. Sreenu, Mayke Leggewie, Mine Altinli, Marlis Badusche, Hanna Jöst, Jessica Börstler, Jonas Schmidt-Chanasit, Esther Schnettler

**Affiliations:** 1 Bernhard-Nocht-Institute for Tropical Medicine, 20359 Hamburg, Germany; 2 School of Basic and Biomedical Sciences, Department of Biomedical Sciences, University of Health and Allied Sciences, PMB 31, Ho, Ghana; 3 Faculty of Mathematics, Informatics and Natural Sciences, University of Hamburg, 20148 Hamburg, Germany; 4 MRC-University of Glasgow Centre for Virus Research, Glasgow, G61 1QH UK; 5 German Center for Infection Research (DZIF), Partner Site Hamburg-Luebeck-Borstel-Riems, Hamburg, Germany

**Keywords:** Mosquito-specific viruses, Interference, Mesonivirus, Negevirus, Arbovirus, RNAi, Culex, Aedes

## Abstract

**Background:**

Mosquito-specific viruses (MSVs) comprise a variety of different virus families, some of which are known to interfere with infections of medically important arboviruses. Viruses belonging to the family* Mesoniviridae* or taxon Negevirus harbor several insect-specific viruses, including MSVs, which are known for their wide geographical distribution and extensive host ranges. Although these viruses are regularly identified in mosquitoes all over the world, their presence in mosquitoes in Germany had not yet been reported.

**Methods:**

A mix of three MSVs (Yichang virus [*Mesoniviridae*] and two negeviruses [Daeseongdong virus and Dezidougou virus]) in a sample that contained a pool of *Coquillettidia richiardii* mosquitoes collected in Germany was used to investigate the interaction of these viruses with different arboviruses in *Culex*-derived cells. In addition, small RNA sequencing and analysis of different mosquito-derived cells infected with this MSV mix were performed.

**Results:**

A strain of Yichang virus (*Mesoniviridae*) and two negeviruses (Daeseongdong virus and Dezidougou virus) were identified in the *Cq. richiardii* mosquitoes sampled in Germany, expanding current knowledge of their circulation in central Europe. Infection of mosquito-derived cells with these three viruses revealed that they are targeted by the small interfering RNA (siRNA) pathway. In* Culex*-derived cells, co-infection by these three viruses had varying effects on the representative arboviruses from different virus families (*Togaviridae*: Semliki forest virus [SFV];* Bunyavirales*: Bunyamwera orthobunyavirus [BUNV]; or *Flaviviridae*: Usutu virus [USUV]). Specifically, persistent MSV co-infection inhibited BUNV infection, as well as USUV infection (but the latter only at specific time points). However, the impact on SFV infection was only noticeable at low multiplicity of infection (MOI 0.1) and at specific time points in combination with the infection status.

**Conclusions:**

Taken together, these results are important findings that will lead to a better understanding of the complex interactions of MSVs, mosquitoes and arboviruses.

**Graphical Abstract:**

**Figure:**
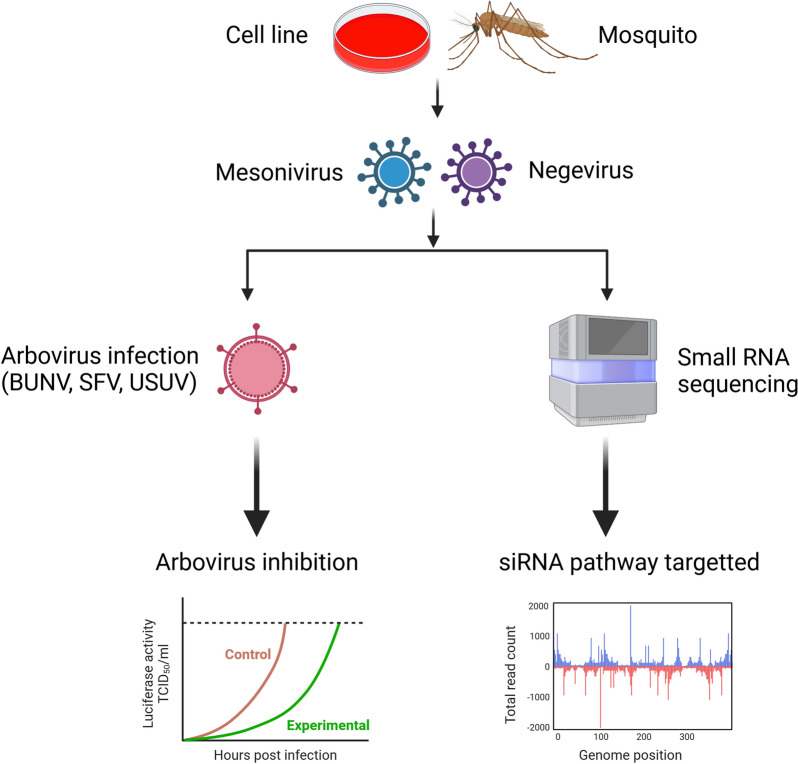

**Supplementary Information:**

The online version contains supplementary material available at 10.1186/s13071-023-05985-w.

## Background

Mosquitoes are responsible for the transmission of human pathogenic arthropod-borne viruses (arboviruses) but can also be infected with mosquito-specific viruses (MSVs). In contrast to arboviruses, MSVs cannot infect vertebrates. MSV is a general term for viruses that belong to a variety of virus families, including some important arbovirus families such as *Togaviridae* and *Flaviviridae* [1]. Similar to arboviruses, MSVs infect and replicate in mosquitoes and derived cells, and the subsequent infection is normally persistent, characterized by continuous virus production but no obvious cytopathic effect or pathology. Due to this lack of pathology, the discovery of these viruses and knowledge of their distribution have lacked strongly behind those on arboviruses. However, over the last decade, next-generation sequencing and bioinformatic analyses have resulted in a steady increase in the discovery of and research on MSVs. One factor increasing interest in MSVs is that some have been reported to interfere with arbovirus infections; however, most of these studies focused on MSVs that are closely related to arboviruses (e.g. *Togaviridae*, *Bunyavirales* and *Flaviviridae*). In addition, either acute or persistent MSV infections are generally used in studies, but rarely are both infection phases directly compared for their effect on arbovirus infections [reviewed in 1, 2]. Interactions of MSVs and arboviruses are normally investigated as single infections; however, data suggest that multiple MSVs co-infect a single mosquito or derived cells [3–9].

Despite the increase in research on MSV–arbovirus interactions, little is known about their interactions with the mosquito host, including the host’s innate immune response. The sequence-specific RNA breakdown mechanism, called RNA interference (RNAi) is the major antiviral response in mosquitoes. It can be divided into several pathways, depending on the key proteins and the produced small RNA (sRNA) molecules. The small interfering (si)RNA pathway has been found to act in an antiviral manner against all viruses tested so far. This pathway is triggered by long double-stranded (ds)RNA, which in turn is cut into 21-nucleotide (nt) siRNAs by Dicer-2. Following the incorporation of the siRNAs into a multiprotein complex, Ago2 protein binds one of the strands and then uses it as a guide to find complementary RNA, followed by RNA degradation. In contrast, the P-element-induced wimpy testis (PIWI)-interacting RNA (piRNA) pathway is independent of Dicer but includes Ago3 and several PIWI proteins. Hallmarks of the piRNA pathway are piRNAs in the size range of 24–30 nt, with specific molecular characteristics due to a ping-pong amplification pathway. Ping-pong-produced piRNAs have a sequence bias (A10 bias for sense and U1 bias for antisense piRNA-sized sRNAs), and the sense and antisense sRNAs have an overlap of 10 nt [10, 11]. Production of virus-specific sRNAs in mosquitoes and derived cells for a variety of MSVs have been reported in past studies; however, these focused nearly exclusively on long-term established persistent MSV infection in mosquito cell cultures or field-caught mosquitoes [3, 6, 7, 12, 13]. In addition, the mosquito immune responses have mostly been investigated in *Aedes* spp. [14] even though other mosquito species, specifically *Culex* spp. have been shown to harbor a variety of MSVs and are known vectors of several important arboviruses (e.g. West Nile virus, Usutu virus, Sindbis virus) [15, 16].

In this study, we have isolated viruses from a mosquito homogenate (derived from a pool of three *Coquillettidia richiardii* mosquitoes). Next-generation sequencing showed that the isolate was a mix of three viruses belonging to *Mesoniviridae* (MesV; Yichang virus [YicV]) and the taxon Negevirus (NeV; namely Dezidougou virus [DeziV] and Daeseongdong virus [DaesV]). Given that we were not able to separate out these viruses and that they are found in close proximity in nature, we studied the effects of co-infection by these three MSVs on the mosquito RNAi response and on arboviruses belonging to different virus families.

## Methods

### Cell lines

Hsu and CT cells originally derived from *Culex quinquefasciatus* and *Culex tarsalis,* respectively, were used as experimental material (gifts from R. van Rij, Radboud University, Nijmegen, the Netherlands). These cell lines are known to harbor several MSV infections [7]. Aag2 and C6/36 cells were received from A. Kohl (University of Glasgow Centre for Virus Research [CVR], Glasgow, UK) and are *Aedes aegypti-* and *Aedes albopictus-*derived cells, respectively. Aag2 harbor previously reported MSVs, in contrast to the C6/36 cells, which are MSV-free [3, 17]. All mosquito-derived cells (Aag2, C6/36, CT and Hsu) were grown at 28 °C and no additional CO_2_ in Leibovitz's L15 medium (PAN-Biotech GmbH, Aidenbach, Germany) supplemented with 10% FCS (PAN-Biotech GmbH), 1× penicillin/streptomycin (Gibco® Thermo Fisher Scientific, Waltham, MA, USA), and 10% tryptose broth (Sigma-Aldrich Chemie GmbH, Taufkirchen, Germany). Vero cells (*Cercopithecus aethiops*; supplied by the ATCC as Vero CCL-81; ATCC, Manassas, VA, USA), BHK-21 cells (*Mesocricetus auratus*; present in the laboratory for a long time) and BSRT7 cells (BHK-21 cells stably expressing the T7 RNA polymerase [18]) were maintained at 37 °C in 5% CO_2_. Vero cells were grown in DMEM medium (PAN-Biotech GmbH) supplemented with 10% FCS, 1× penicillin/streptomycin and 10% tryptose broth. BHK-21 and BSRT7 cells were grown in GMEM medium (Life Technologies, Thermo Fisher Scientific) supplemented with 10% FCS, 1× penicillin/streptomycin and 10% tryptose broth.

Hsu and CT cells persistently co-infected with YicV/DeziV/DaesV were established. Briefly, 1 × 10^6^ cells were seeded in a 6-well plate and co-inoculated with YicV/DeziV/DaesV. Cells were checked regularly and passaged twice a week. Cell lines that showed a lack of viral clearance after three passages were defined as persistently infected cells. The presence of the viruses was verified regularly over the course of the experiments (passage 3 to passage 20) as follows: RNA was isolated from cells using TRIzol (Thermo Fisher Scientific), following the manufacturer’s protocol; then cDNA was produced from 1.5 µg total RNA, using M-MLV (a recombinant DNA polymerase; Promega, Madison, WI, USA) with random hexamers (Promega), and the presence of YicV, DeziV and DaesV were verified by GoTaq PCR (Promega) using virus-specific primer pairs (Additional file 1: Table S1).

### Virus detection and isolation

The virus mix was isolated from one MeSV generic PCR-positive mosquito pool (consisting of 3 *Cq. richiardii*). The mosquitoes were collected on the island of Kühkopf in the state of Hesse in Germany during the 2014 field season using dry ice-baited EVS traps as well as gravid traps, BG-Sentinel traps, sweeping nets and hand-held aspirators. Mosquitoes were pooled together by species and homogenized in 1 ml of cell culture medium (high-glucose Dulbecco’s modified Eagles’s medium). RNA was extracted from the homogenates (400 µl) using the RTP Pathogen Kit (Stratec Biomedical AG, Birkenfeld, Germany), followed by a generic MeSV PCR using the SuperScript® III One-Step RT-PCR System with Platinum^®^ (Life Technologies, Thermo Fisher Scientific) with corresponding primers (Additional file 1: Table S1: MeSV-FW & RV) [19]. The isolation was performed on C6/36 cells, according to established protocols [19, 20]. Briefly, the 1-ml mosquito pool homogenate was clarified by centrifugation, followed by the addition of the cleared supernatant onto C6/36 cells and incubation at 28 °C.

Following observation of cytopathic effects (CPE), the supernatant was passaged again on C6/36 cells to produce a virus working stock (named 8345), followed by virus discovery with a next-generation sequencing approach.

### Viruses

Usutu virus (USUV 491; accession number KY426758; *Flaviviridae*) [21] working stock was produced in Vero cells, and the virus titer was determined in Vero cells by the TCID_50_ assay.

Semliki Forest virus (SFV; *Togaviridae*) expressing Nano luciferase (SFV6-2SG-NLuc) was produced in BHK-21 cells by transfection of the corresponding plasmid pCMV-SFV6-2SG-Nluc as previously described [17, 22]. This plasmid contains a cytomegalovirus (CMV) promoter expressing reporter virus complementary DNA (cDNA) based on the SFV6 clone and expresses Nano luciferase through a duplicate subgenomic promoter.

Similarly, Bunyamwera orthobunyavirus (BUNV; *Bunyavirales*) expressing Nano luciferase (BUNV-Nluc) was produced in BSRT7 cells by co-transfection of three plasmids: pTVT7RBUNM-NL (a part of the BUNV NSm cytoplasmic domain was replaced by the Nluc sequence resulting in chimeric NSm-Nluc fusion protein), pT7riboBUNL(+) and pT7riboBUNS(+) encoding the BUNV antigenome (Additional file 2: Figure S1). Following observation of CPE, the supernatant was transferred to produce virus working stocks. Virus titer was determined, in BHK-21 cells, by the plaque assay with Avicel as previously described [23].

### Next-generation sequencing, virus discovery and phylogenetic analysis

For next-generation sequencing, RNA was isolated from infectious C6/36 cell supernatant (8345) using the Mag-MAX Viral RNA Isolation Kit (Thermo Fisher Scientific) according to the manufacturer’s instructions. This was followed by next-generation sequencing, as previously described [24].

The obtained reads were adapter trimmed using Trim Galore! and mapped against the C6/36 reference transcriptome (GCF_001876365.2) using BWA-MEM2. Unmapped reads were retained and processed using the Trinity method [25]. The constructed sequences were identified using the BLASTN program. To avoid sequence contamination, the protocol was repeated with libraries additionally filtered by mapping either to MeSV or NeVs, respectively. The viral genomes constructed using the Trinity method were used to infer the phylogenetic relation to known MeSV and NeV viruses, respectively, using MEGA X. For MeSV, the concatenated amino acid sequences of the conserved regions of the 3CL_PRO_, RdRp and helicase were used. The inferred maximum likelihood phylogeny was created using the LG + F substitution model (as suggested by MEGA X Model Selection applying default parameters), assuming a gamma distribution and 1000 replicates. Based on this, the paired evolutionary distance (PED) analysis was also performed. For NeVs, the maximum likelihood phylogeny was inferred based on the concatenated amino acid sequences of the conserved regions of the methyltransferase, helicase and RdRp domains, using the LG substitution model and assuming gamma distribution with invariant sites and 1000 replicates.

### sRNA sequencing and β-elimination assay

To investigate the production of YicV-, DeziV- and DaesV-specific sRNAs in CT and Aag2 cells, 8 × 10^5^ cells were seeded in a 6-well plate and co-infected with YicV/DeziV/DaesV. Total RNA was isolated at 24 h post infection (hpi) with TRIzol according to the manufacturer’s protocol, with glycogen as a carrier. Successful infection was verified by PCR using virus-specific primers (Additional file 1: Table S1) with cDNA, produced from 1.5 µg of total RNA with random hexamers and M-MLV. To determine the methylation status of the sRNA (in case of persistently infected CT cells, passage 3), a β-elimination assay was performed, as previously described [22]. In short, TRIzol-isolated total RNA samples were equally divided into two portions, following which 5 µl of 20× borate buffer, 12.5 µl of sodium periodate (or water in the case of the control sample) and 100 µl RNase-free H_2_O were added. After 15 min, 10 µl of glycerol was added and the samples incubated for a further 15 min at room temperature. Ethanol precipitation was then performed with glycogen as a carrier. Subsequently, precipitated, dried pellets were resuspended in borate buffer followed by 90 min incubation at 45 °C. RNA was purified using the Monarch RNA Cleanup kit (50 µg) (New England Biolabs, Inc., Ipswich, MA, USA) according to the manufacturer’s protocol. At least 1 µg of total RNA was sent for sRNA sequencing using an Illumina-based system at BGI (BGI-tech solutions, Hong Kong; BGISEQ-500) as previously described [3].

RNA Workbench 2.0 [26] was used to analyze YicV/DeziV/DaesV-derived sRNA. First, the reads were mapped against the respective reference genome using the BWA software package. Then, uniquely mapped reads were sorted into three separate libraries. The complete set of all 18- to 31-nt reads were counted and plotted against their respective size. All 26- to 30-nt reads were included in the nucleotide versus cycle plot to show nucleotide position biases and used to compute sequence overlaps using the software signature.py [27]. These and all 21-nt reads were used to create bed graphs of genome coverage distribution of reads per base, respectively.

Nucleotide sequences of the identified viruses are available in the NCBI Genbank under the numbers OP576001 (YicV8345), OP576002 (DaesV8345) and OP576003 (DeziV8345). sRNA sequencing data and fastq files are available in the NCBI Sequence Read Archive under BioProject ID PRJNA885760.

### YicV/DeziV/DaesV infection in different cells

Corresponding cells (CT, Hsu, Aag2, Vero and BHK-21) were plated in 24-well plates. To reach approximately 80% confluency on the day of infection, 1.8 × 10^5^ cells/well were plated in the case of Vero, BHK-21 and Aag2 cells. Due to their slower growth, 2.5 × 10^5^ cells/well of CT and Hsu cells were used. After 24 h, the growth media were removed, and virus was added (combined multiplicity of infection [MOI] 1). After 1 h, the medium was removed, and cells were washed with 1× phosphate buffered saline, followed by the addition of the corresponding complete media. Cell pellets were harvested at 72 hpi. RNA was isolated by TRIzol (Thermo Fisher Scientific) according to the manufacturer’s protocol. cDNA was then produced with random hexamers and the M-MLV reverse transcriptase, followed by PCR with virus-specific primer pairs (Additional file 1: Table S1).

### YicV/DeziV/ DaesV quantification method

RNA from infected cells were isolated by TRIzol (Thermo Fisher Scientific) according to the manufacturer’s protocol.

For the quantification of the three viruses in the working stock, RNA was isolated from 140 µl of working stock with the QIAamp Viral Isolation Kit (QIAGEN, Hilden, Germany) according to the manufacturer’s protocol. cDNA was synthesized (to be used in quantitative PCR [qPCR]) using either 1.5 µg of RNA (infected cells) or 14 ul isolated RNA (Qiamp isolation kit), random hexamers and M-MLV reverse transcriptase (Promega) according to the manufacturer’s protocol.

To quantify the relative DeziV, DaesV and YicV load in the different samples, we performed two-step qPCR using cDNA samples, virus-specific primers (Additional file 1: Table S1) and QuantiTect SYBR Green PCR kit (QIAGEN, Germany) according to the manufacturer’s protocol.

All PCR reactions were performed in two technical replicates. The data were analyzed using the LightCycler® 480 PCR platform (Roche Diagnostics, Rotkreuz, Switzerland) and LightCycler 480 software (version 1.5.0 SP4), using the absolute quantification method. Standard curves for each virus genome were created using 10-fold dilutions of the specific PCR products (DNA amplicon), and relative copy numbers were calculated. CP values of > 30 for DeziV,  > 28 for DaesV and > 31 for YicV were determined as cut-off values.

### Effect of YicV/DeziV/DaesV co-infection on arbovirus infection

CT cells or CT cells persistently infected by YicV/DeziV/DaesV were seeded in a 96-well plate with 6 × 10^4^ cells/well. The following day, CT cells were co-infected with YicV/DeziV/DaesV and either SFV6-2SG-Nluc (MOI 1 or MOI 0.1) or BUNV-Nluc (MOI 0.1), respectively. Different MOIs were used according to previously reported susceptibility [28]. As the control, CT cells were singly infected with SFV6-2SG-Nluc (MOI 1, MOI 0.1) or BUNV-Nluc (MOI 0.1), respectively. In addition, CT cells persistently infected with YicV/DeziV/DaesV were infected with SFV6-2SG-Nluc (MOI 1 or MOI 0.1) or BUNV-Nluc (MOI 0.1), respectively.

Nanoluciferase activity of the infected CT cells was determined at different time points. Briefly, cells were lysed with passive lysis buffer, and Nanoluciferase activity was measured using the Nano-Glo® Luciferase Assay System and GloMax luminometer (Promega) according to the manufacturer’s protocol. Three independent experiments in triplicate were performed for each experimental set-up.

For USUV infection, CT cells or CT cells persistently co-infected with YicV/DeziV/DaesV were seeded in a 12-well plate with 4 × 10^5^ cells/well. The next day, CT cells were co-infected with YicV/DeziV/DaesV and USUV (MOI 10). As a control, CT cells were singly infected with USUV (MOI 10). In addition, CT cells persistently infected with YicV/DeziV/DaesV were infected with USUV (MOI 10). The supernatant (200 µl) was collected at different time points (0, 6, 10, 24, 48 and 72 hpi), and the USUV titer was determined by the TCID_50_ assay in Vero cells. Three independent experiments in triplicate were performed.

To ensure reproducibility of the results for CT cells persistently infected with YicV/DeziV/DaesV, independent biological replicates were performed using cells from different passages between the 12th to the 20th passage following virus introduction.

### Statistical analysis

Data generated from the MSV-arbovirus infection experiments were analyzed and graphs prepared using GraphPad Prism version 7.00 (GraphPad Software Inc., San Diego, CA, USA). Statistical analyses were performed using an unpaired, two-tailed t-test. A *P*-value < 0.05 was considered to be significant.

## Results

### Isolation of a mesonivirus and negeviruses mix from a mosquito homogenate from Germany

A homogenate from a mosquito pool of three *Cq. richiardii*, captured together at the same collection site during an earlier study [19], caused a strong CPE in C6/36 cells.

Collected supernatant (referred to as 8345) was used for in-depth virus discovery analysis by next-generation sequencing. De novo transcript assembly suggested the presence of three positive single-stranded RNA viruses: one mesonivirus (MeSV) and two negeviruses (NeVs).

**Fig. 1:**
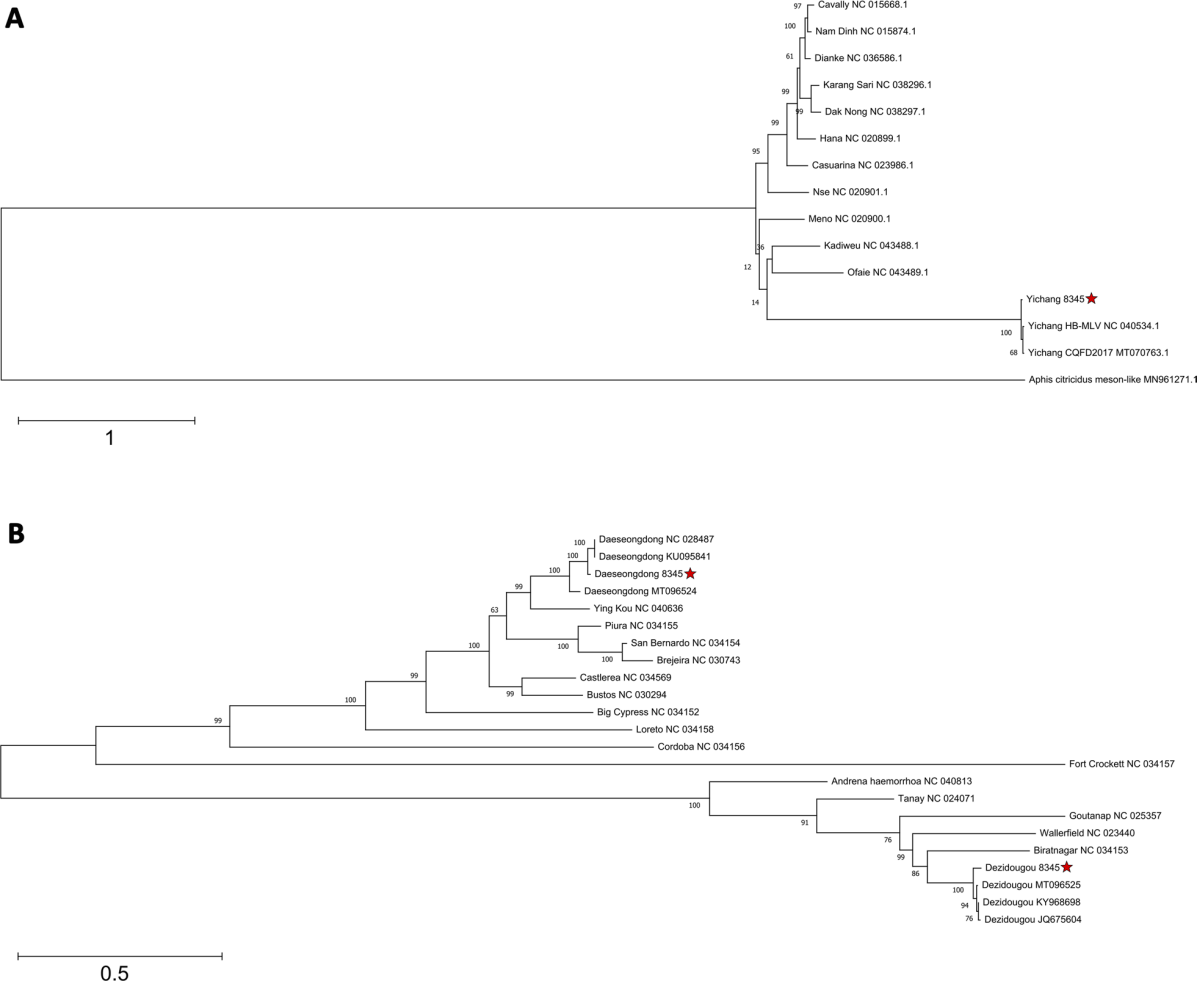
Maximum likelihood phylogeny of MeSV and NeV samples. The phylogenetic inference of MeSV was based on a concatenated amino acid alignment of the conserved regions of the 3CLpro, RdRp and helicase domains (**a**). The phylogenetic inference of NeV was based on a concatenated amino acid alignment of the conserved regions of the methyltransferase, helicase and RdRp domains (**b**). The virus species identified in this study are marked with a red star. The bootstrap values represent 1000 replicates. The trees are drawn to scale, with branch lengths measured in the number of substitutions per site. MeSV,* Mesoniviridae*; NeV, taxon Negevirus

The MeSV sequence identified in the pool was identified as YicV with a global nucleotide sequence identity of 96.5% to the closest variant (MT070763.1). One of the NeV was identified as Daeseongdong virus (DaesV), with a global nucleotide sequence identity of 90.8% to the closest known strain (KU095841.1). The other was identified as DeziV, with an identity of 83.1% to the closest Genbank hit (JQ675604.1). The YicV, DaesV and DeziV variants identified clustered closely with their known relatives and were therefore considered to be of the same species (Fig. 1).

Various attempts to separate the three viruses (YicV, DaesV, DeziV) by different methods (plaque purification using Tragacanth, dilution assays or a combination of both assays) were unsuccessful. Therefore, the following experiments were performed with a virus working stock harboring all three virus strains.

### Susceptibility of cell lines to YicV/DeziV/DaesV and production of persistently infected *Culex*-derived cells

**Fig. 2:**
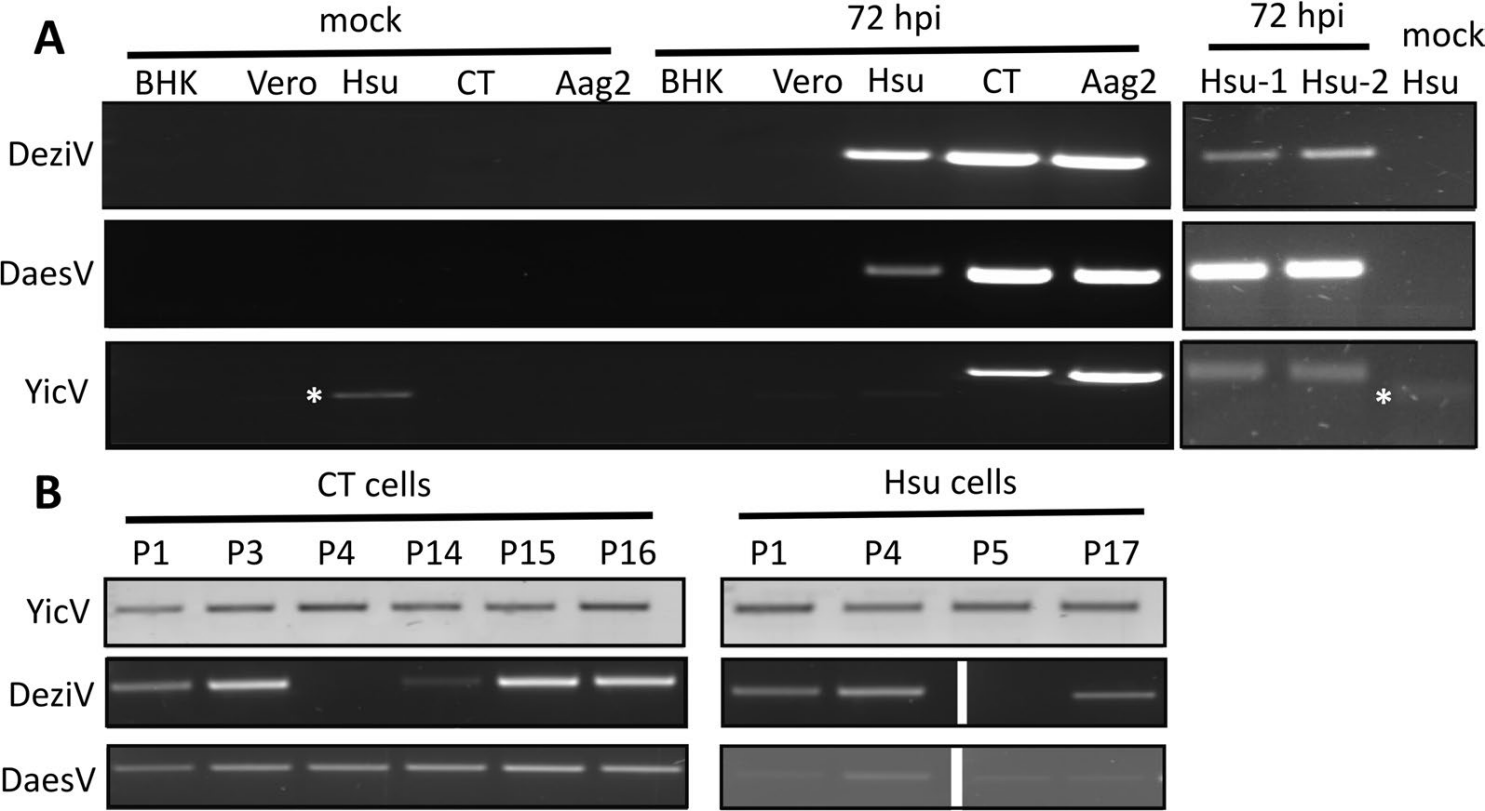
Mosquito-derived and mammalian cell lines co-infected with YicV/DeziV/DaesV.** a** Hsu (*Culex quinquefasciatus*), CT (*Culex tarsalis*), Aag2 (*Aedes aegypti*), Vero (*Chlorocebus* sp.) and BHK (*Mesocricetus auratus*) cells infected with the YicV/DeziV/DaesV mix (representative data of 2 independent repeats; except for Hsu cells). Right panel shows two additional repeats for Hsu-infected cells (Hsu-1 and Hsu-2, respectively).** a**,** b** The presence of YicV, DeziV and DaesV was detected by reverse transcription and virus-specific primers at 72 hpi (**a**) or at several passages for persistently infected CT and Hsu cells (**b**). The asterisk indicated an unspecific PCR band (verified by Sanger sequencing) in Hsu cells. DaesV, Daeseongdong virus (taxon Negevirus); DeziV, Dezidougou virus (taxon Negevirus); hpi, hours post infection; P, passage; YicV, Yichang virus (family* Mesoniviridae*)

As the YicV/DeziV/DaesV mix derives from the homogenate of the same small mosquito pool, we determined if YicV/DeziV/DaesV could successfully co-infect different mosquito-derived cell cultures belonging to *Aedes* and *Culex* species (Aag2, Hsu and CT cells) and mammalian cells (Vero and BHK-21 cells). Infection experiments were performed, followed by RNA isolation at 72 hpi and reverse transcription-PCR (RT-PCR). The presence of all three viruses in the mosquito cells was verified by RT-PCR (Fig. 2a). The amount of DeziV and DaesV was similar in Aag2 and CT cells, but the amount of YicV was found to be at least 600-fold higher in infected Aag2 cells compared to CT cells (Additional file 3: Table S2). The presence of YicV in Hsu cells could be detected in two of the three repeats. In mock-infected Hsu cells, YicV-specific RT-PCR showed repetitively an unspecific PCR product (based on Sanger sequencing results; data not shown).

The lack of any of the three viruses in the tested mammalian cells (BHK and Vero) implicates their insect-specific characteristics.

To determine if YicV/DeziV/DaesV could induce a persistent infection in *Culex*-derived cells, Hsu and CT cells were infected and passaged at least 5 times. The CPE was observed in the initial infection in Hsu and CT cells (at around 24 hpi), but both cell types successfully recovered at around 72 hpi. After several passages, the presence of all three viruses was detected by RT-PCR (Fig. 2b). To determine possible changes in the ratios of the viruses in the persistently infected cells, relative viral copy numbers were determined in several of the passages (Additional file 4: Table S3). The DeziV to DaesV ratio was < 1 in selected (CT cells: passage 16 and 20; Hsu cells: passage 4, 5 and 17) and persistently infected cells tested (CT and Hsu), except for passages 3 and 4 in CT cells.

### Effect of YicV/DeziV/DaesV on arbovirus infection in *Culex*-derived cells

**Fig.3:**
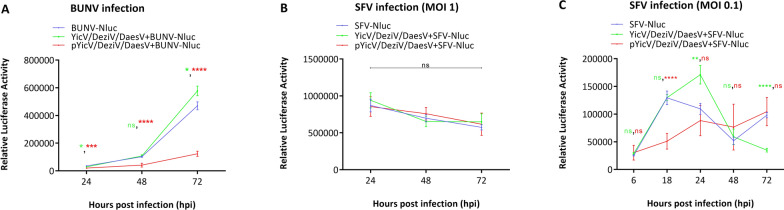
Effect of YicV/DeziV/DaesV infection status on Semliki Forest or Bunyamwera orthobunya arbovirus infections in CT (*Culex tarsalis*)-derived cells. CT cells were infected with BUNV-NLuc (BUNV expressing NLuc; MOI 0.1) (**a**) or SFV6-2SG-Nluc (SFV-Nluc, SFV expressing Nluc; MOI 1 or MOI 0.1) (**b, c**), either single, acutely co-infected with YicV/DeziV/DaesV (YicV/DeziV/DaesV + SFV-Nluc; MOI 1, MOI 0.1 or YicV/DeziV/DaesV + BUNV-NLuc; MOI 0.1) or using cells persistently infected with YicV/DeziV/DaesV (pYicV/DeziV/DaesV + SFV-Nluc; MOI 1, MOI 0.1 or pYicV/DeziV/DaesV + BUNV-NLuc; MOI 0.1). Cells were lysed at the indicated hours post infection (hpi) and luciferase activity was measured. Results of three independent experiments performed in technical triplicates are presented. Mean values with standard error of the mean are shown. Significance was tested via unpaired t-test. BUNV infection: acute infection (24 hpi: *t* = 2.448, *P = 0.0263; 72 hpi: *t* = 2.389, *P = 0.0296); persistent infection (24 hpi: *t* = 5.180, ***P = 0.0002; 48 hpi: *t* = 9.981, ****P < 0.0001; 72 hpi: *t* = 11.69, ****P < 0.0001). SFV infection MOI 0.1: acute infection (24 hpi: *t* = 3.143, **P = 0.0063; 72 hpi: *t* = 10.59, ****P < 0.0001); persistent infection (18 hpi: *t* = 5.962, ****p < 0.0001). BUNV, Bunyamwera orthobunya virus; DaesV, Daeseongdong virus; DeziV, Dezidougou virus; MOI, multiplicity of infection; Nluc, Nano luciferase; ns, not significant; YicV, Yichang virus; SFV Semliki forest virus

A growth kinetics study was performed to investigate the effect of YicV/DeziV/DaesV on arboviruses in *Culex*-derived cells. Three arboviruses were chosen as representatives of important arbovirus families/orders (*Flaviviridae*, *Bunyavirales* and *Togaviridae*) that are also known to infect* Culex* mosquitoes: SFV (*Togaviridae*), BUNV (*Bunyavirales*) and USUV (*Flaviviridae*). The type of cells used and MOI were based on previous results (SFV and BUNV [29]; USUV, data not shown). CT cells were co-infected with YicV/DeziV/DaesV and SFV6-2SG-Nluc or BUNV-Nluc luciferase reporter viruses. Also, CT cells persistently infected with YicV/DeziV/DaesV were included and again infected with either SFV6-2SG-Nluc or BUNV-Nluc, respectively. As a control, CT cells infected only with the corresponding arbovirus (SFV or BUNV) were included. Luciferase expression was determined at different time points. In the case of SFV infection at high MOI (MOI 1), no increase of luciferase over time was observed. Generally, there were no differences between single infected CT cells and YicV/DeziV/DaesV-infected CT cells, independent of the acute or persistent infection status (Fig. 3b). However, in case of SFV infection at a low MOI (0.1), single and co-infection resulted in a rapid increase of luciferase and a peak at 18 or 24 hpi, respectively. Afterwards, a decrease in luciferase was detected for both infections. SFV infection in persistently infected cells showed a slower but steady increase over time (Fig. 3c), resulting in lower relative luciferase activity in persistently infected cells at 18 hpi, but not at later time points. Depending on the time post infection, co-infection resulted in higher relative luciferase (24 hpi) and lower luciferase at 72 hpi. In summary, no general effect on SFV infection could be detected as the effect of YicV/DeziV/DaesV infection on SFV depended on the time point of detection, infection status (acute vs persistent) and initial SFV infection dose.

For BUNV infection, no difference or only a small difference between the co-infection and single infection was observed (Fig. 3). In contrast, lower relative luciferase activity was observed for BUNV in CT cells persistently infected with YicV/DeziV/DaesV compared with BUNV-single infected control cells.

Similar effects for SFV (high MOI 1) and BUNV were observed when *Cx. quinquefasciatus*-derived Hsu cells were used instead of CT cells (Additional file 5: Figure S2).

Taken together, YicV/DeziV/DaesV infection showed some effect on SFV in the case of low MOI but none at all at high MOI. In contrast, persistent YicV/DeziV/DaesV infection had an inhibitory effect on BUNV infection but not in acutely co-infected cells. The DeziV to DaesV ratio differed between the persistently and acutely infected CT cells (Additional file 6: Table S4, DeziV < DaesV in persistently infected cells and DeziV > DaesV in acutely infected cells).

**Fig. 4:**
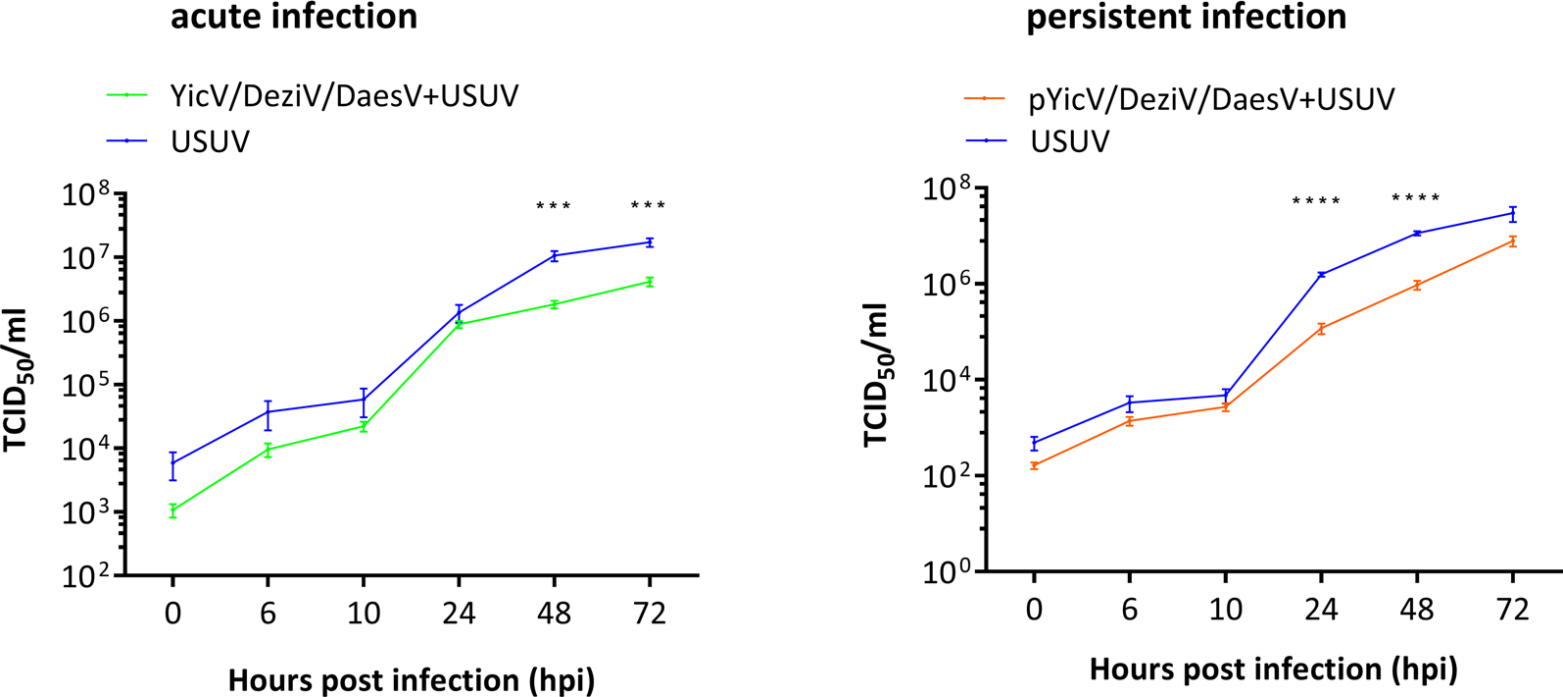
Effect of YicV/DeziV/DaesV infection status on Usutu arbovirus infections in CT (*Culex tarsalis*)-derived cells. CT cells singly infected with Usutu virus (USUV, MOI 10) were compared to cells either acutely co-infected with YicV/DeziV/DaesV and USUV (YicV/DeziV/DaesV + USUV; MOI 10) or to USUV infection in cells persistently infected with YicV/DeziV/DaesV (pYicV/DeziV/DaesV + USUV; MOI 10). Supernatants were collected for USUV titration via TCID_50_ at 0, 6, 10, 24, 48 and 72 hpi for 3 independent experiments in technical triplicates. Mean values with standard error of the mean are shown. Significance was tested via the unpaired t-test. Acute infection (48 hpi: *t* = 4.507, ***p = 0.0004; 72 hpi: *t* = 4.706, ***p = 0.0002); persistent infection (24 hpi = *t* = 9.487, ****p < 0.0001; 48 hpi: *t* = 8.959, ****p < 0.0001). DaesV, Daeseongdong virus; DeziV, Dezidougou virus; MOI, multiplicity of infection; TCID_50_, median tissue culture infectious dose assay; USUV Usutu virus; YicV, Yichang virus

To investigate the effect of YicV/DeziV/DaesV on USUV infection, CT cells were either co-infected with YicV/DeziV/DaesV and USUV or singly infected with USUV. In addition, CT cells persistently infected with YicV/DeziV/DaesV were infected with USUV. The titer of USUV in the supernatant at different time points was determined by TCID_50_. Reduced USUV titer was observed in acute co-infections and persistently infected CT cells, but only at certain time points: 48 and 72 hpi in co-infections and 24 and 48 hpi in cells persistently infected with YicV/DeziV/DaesV (Fig. 4).

### YicV, DeziV, DaesV are targeted by the RNAi response in co-infected mosquito-derived cells

**Fig. 5:**
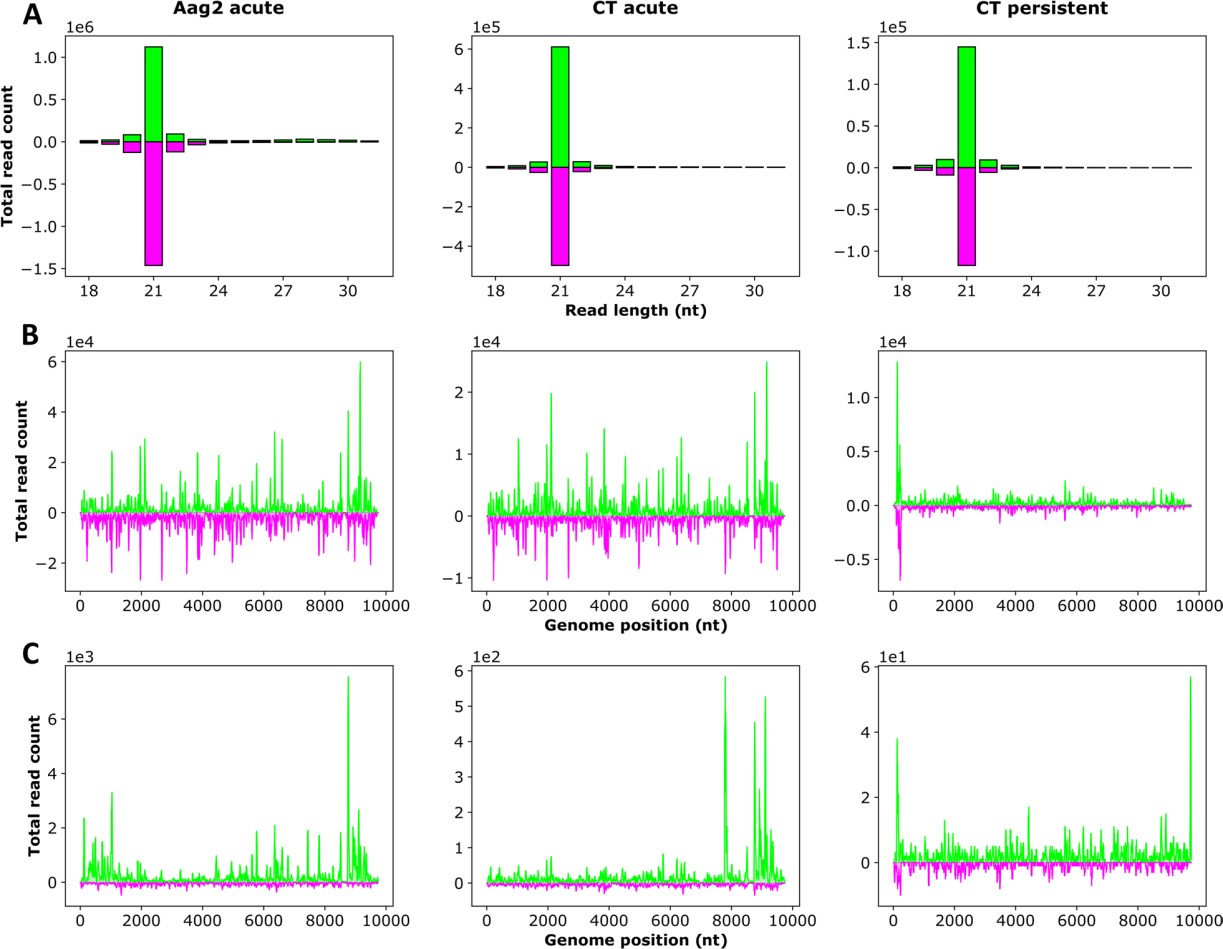
Production of DaesV-specific sRNAs in CT (*Culex tarsalis*) and Aag2 (*Aedes aegypti*) cells acutely infected with YicV/DeziV/DaesV or CT cells persistently infected with YicV/DeziV/DaesV. For acute infection, cell were infected with the DaesV/DeziV/YicV mix, and total RNA was isolated at 24 h post-infection. RNA of persistently infected CT cells (passage 3) were isolated, followed by β-elimination treatment (Additional file 8: Figure S4) and control (CT persistent).** a** The absolute frequency of sRNAs ranging in length from 18 to 31 nt that were mapped to the virus genome/antigenome. **b** The distribution of 21-nt-long sRNA to the indicated virus genome/antigenome. **c** The the distribution of 26- to 30-nt-long sRNAs (piRNA-sized) to the virus genome/antigenome is shown. Positive values (green) represent sense reads, negative values (purple) represent antisense reads.* Y*-scale values give the read counts, with the scale values mentioned above the graph. Representative results of the acute infection of a duplicate (Additional file 7: Figure S3). DaesV, Daeseongdong virus; DeziV, Dezidougou virus; nt, nucleotide; sRNAs, small RNAs; YicV, Yichang virus

**Fig. 6:**
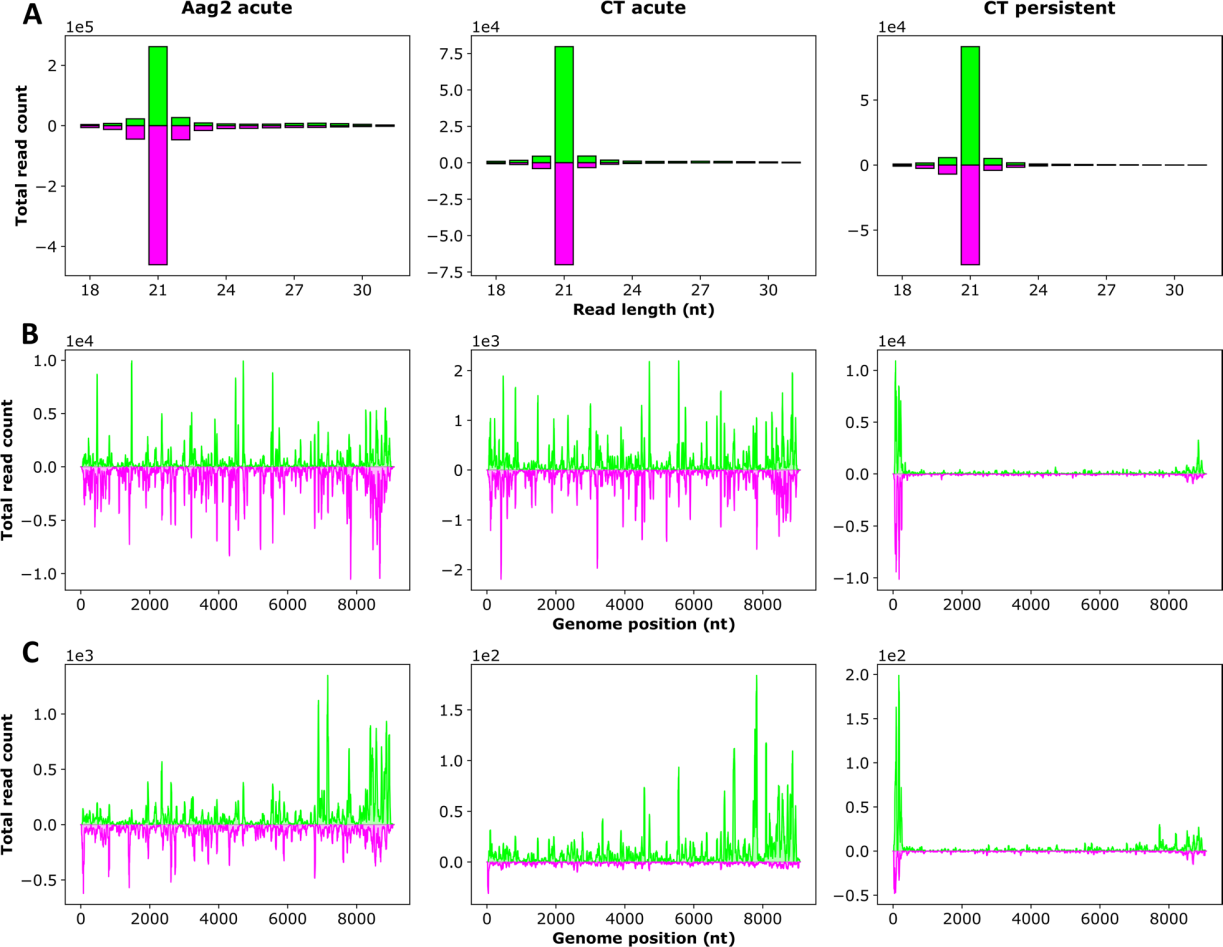
Production of DeziV-specific sRNAs in CT (*Culex tarsalis*) and Aag2 (*Aedes aegypti*) cells acutely infected with YicV/DeziV/DaesV or CT cells persistently infected ith YicV/DeziV/DaesV. For acute infection, cells were infected with the DaesV/DeziV/YicV mix and total RNA was isolated at 24 h post-infection. RNA of persistently infected CT cells (passage 3) were isolated, followed by β-elimination treatment (Additional file 8: Figure S4) and control (CT persistent).** a** The absolute frequency of sRNAs ranging in length from 18 to 31 nt that were mapped to the virus genome/antigenome. **b** The distribution of 21-nt-long sRNA to the indicated virus genome/antigenome. **c** The distribution of 26- to 30-nt-long sRNAs (piRNA-sized) to the virus genome/antigenome. Positive values (green) represent sense reads, negative values (purple) represent antisense reads.* Y*-scale values give the read counts, with the scale values mentioned above the graph. Representative results of the acute infection of a duplicate (Additional file 7: Figure S3). DaesV, Daeseongdong virus; DeziV, Dezidougou virus; nt, nucleotide; sRNAs, small RNAs; YicV, Yichang virus

**Fig. 7:**
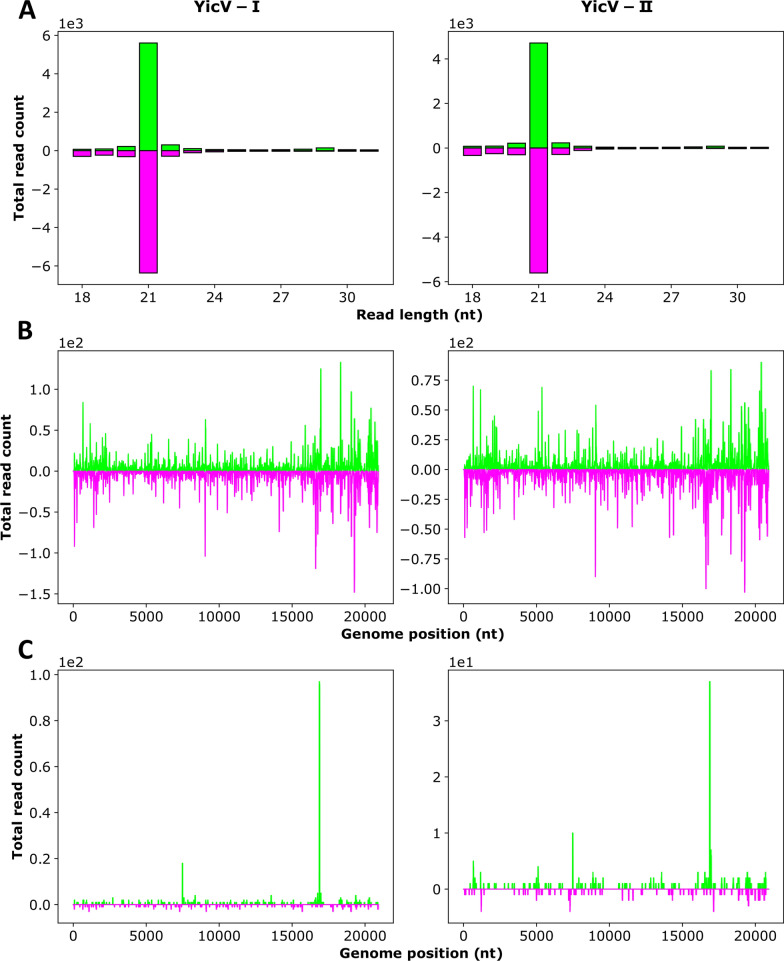
Production of YicV-specific small RNAs in YicV/DeziV/DaesV acutely infected Aag2 (*Aedes aegypti*) cells. Cells were infected with the DaesV/DeziV/YicV mix and total RNA was isolated at 24 h post infection.** a** The absolute frequency of sRNAs ranging in length from 18 to 31 nt that were mapped to the virus genome/antigenome. **b** The distribution of 21-nt-long sRNA to the indicated virus genome/antigenome. **c** The distribution of 26- to 30-nt-long sRNAs (piRNA-sized) to the virus genome/antigenome. Positive values (green) represent sense reads, negative values (purple) represent antisense reads.* Y*-scale values give the read counts, with the scale values mentioned above the graph. Results of two independent experiments are shown (I and II). DaesV, Daeseongdong virus; DeziV, Dezidougou virus; nt, nucleotide; sRNAs, small RNAs; YicV, Yichang virus

To investigate if all three co-infecting viruses (YicV/DeziV/DaesV) are targeted by the RNAi response in different mosquito cells, we analyzed the sRNAs in YicV/DeziV/DaesV-infected CT and Aag2 cells. Total RNA was isolated at 24 hpi, and sRNAs were sequenced and mapped to the above-established genome sequences of DaesV (Fig. 5; Additional file 7: Figure S3), DeziV (Fig. 6; Additional file 7: Figure S3) and YicV 8345 (Fig. 7).

#### siRNA production

Small RNAs derived from DaesV and DeziV were detected in both Aag2 and CT cells. YicV-specific sRNAs were only detectable in Aag2 cells but not in CT cells. Although YicV RNA was observed by RT-PCR in both cell lines, the copy numbers of YiCV were at least 600-fold higher in Aag2 cells than in CT cells (Additional file 3: Table S2). In these cell lines, the viruses primarily induced the production of 21-nt-long siRNAs without pronounced bias toward specific genomic regions (Figs. 5–7a, b; Additional file 7: Figure S3A, B) or bias for the virus genome/antigenome (Additional file 9: Table S5). To investigate if sRNAs derived from one Negevirus could target the co-infecting other negeviruses, corresponding analysis was performed. For this, sRNAs (18–32 nt) produced in response to DaesV infection were mapped to the genome and antigenome of DeziV and vice versa (Additional file 10: Figure S5). The results show only a handful of cross-targeting sRNAs.

#### piRNA production

DaesV and DeziV also induced piRNA-sized sRNAs (26–30 nt) in both cell lines, while YicV only induced a small amount of these piRNA-sized sRNAs in Aag2 cells (Figs. 5–7a, c; Additional file 7: Figure S3A, C). For DeziV, these piRNA-sized sRNAs showed a slight U1 bias in both cell lines. DaesV-specific piRNA-sized sRNAs, only exhibited a U1 bias in Aag2 cells but not in CT cells. Neither DeziV- nor DaesV-specific piRNA-sized sRNAs showed an overlap signature of the sense and antisense sRNAs (Additional file 11: Figure S6; Additional file 12: Figure S7), thereby lacking several of the ping-pong produced piRNA specific features. In addition to the YicV/DeziV/DaesV-specific small RNAs, previously reported virus-specific small RNAs of the MSVs known to be present in the Aag2 cells: Namely Phasi-Charoen-like virus (PCLV) and cell fusing agent virus (CFAV) [17, 29] as well as Merida virus (MERDV) [6, 7] in CT cells, were detected(data not shown).

#### sRNA production in persistently co-infected cells

Differences in virus-derived sRNAs (vsRNA) production and mapping were observed between acute and persistent infection in CT cells. In the persistently infected cells (passage 3), the majority of the DaesV- and DeziV-specific siRNAs mapped to the last few hundred nucleotides of the 3′ and 5′ ends of the genome, with the 5′ end showing an even higher mapping than the 3′ end. This also applies to DeziV- and DaesV-specific piRNA-sized sRNAs (Figs. 5, 6). The ratio of piRNA-sized to siRNA-sized sRNAs was decreased in persistently co-infected CT cells for DeziV and DaesV-specific small RNAs, compared to the acute infection (Additional file 9: Table S5).

#### Biological activity of produced siRNAs

Small RNAs that were bound by Ago2 are methylated at their 3′ end. To determine the methylation status of the produced vsRNAs in persistently co-infected cells, total RNA of CT cells persistently infected with YicV/DeziV/DaesV was isolated. Prior to sRNA sequencing, the β-elimination assay was performed using one-half of each sample; the other half was kept as control to analyze the methylation status of the sRNAs. With the exception of a reduction in vsiRNAs in the β-eliminated cells compared to the control, no major differences could be detected (Additional file 9: Table S5; Figs. 5 and 6; Additional file 8: Fig. S4). Only DeziV-specific small RNAs showed a strong increase in the ratio of genomic to antigenomic reads of piRNA-sized sRNAs in case of β-elimination treatment (Additional file 9: Table S5).

## Discussion

In this study, we have characterized a mixture of three MSVs, consisting of one MeSV (YicV) and two NeVs belonging to distinct NeV groups (DaesV [Nelopivirus] and DeziV [Sandewavirus]) [30], isolated from a mosquito pool from Germany. Our results expand current the knowledge of the circulation of these MSVs in central Europe. The presence and isolation of different MeSVs have been reported in other countries in a variety of mosquito species, proving their wide range of hosts and geographic range. Similarly, NeVs have been isolated from various mosquito genera around the world, and both MeSV and NeV are known to infect important mosquito vector species for arboviruses [1, 31, 32].

The presence of these three distinct MSVs in the homogenate of a pool of only three *Cq. richiardii* mosquitoes and current inability to separate them by commonly used methods (e.g. plaque purification and dilution assays) suggests either a positive interaction or at least a non-inhibitory interaction between these viruses in the C6/36 cell line used in this study. As no separate homogenates of the three *Cq. richiardii* mosquitoes were available, it is impossible to determine if all three viruses were initially present in one mosquito. However, the presence of at least two different MSVs in mosquitoes or derived cell lines has been repetitively reported in the past, thereby supporting the notion of the regular occurrence of co-infections of MSVs in mosquitoes in nature. In particular, co-infection of NeVs and other MSV families have been repetitively reported from mosquitoes (e.g. Eilat virus isolated in Israel or Agua Salud virus isolated in Panama), as have the difficulties or inability to separate these different MSVs [3, 5–9, 33]. It is not known if the reported co-infection of NeVs with other MSVs is due to unknown benefits of NeVs in the case of co-infection or whether it is just accidental due to their wide distribution and broad host range.

Previous studies showed that other NeVs [34] and YicV could inhibit several arboviruses in mosquito-derived cells. In particular, YicV isolated in China is highly homologous to the YicV variant used in our study and showed a strong effect on several flaviviruses (DENV-2 and ZIKV) but not on another flavivirus (Japanese encephalitis virus [JEV]) [35]. In contrast, Negev virus, which is associated with the inhibition of a variety of arboviral alphaviruses, including SFV [34], shares only some homology to the two NeVs present in the MSV mix used in our study.

In our study, some conditions resulted in inhibition and in others the MSV mix had no effect on the different arboviruses tested in *Culex*-derived cells. The persistent MSV mix inhibited BUNV infection by about four-fold. At certain time points, an inhibition effect, although smaller, was also observed for USUV. In contrast, no effect on SFV infection with a high MOI (1 for CT cells and 10 for Hsu cells) was observed for any of the tested conditions. However, the effects on SFV infection were observed in the case of a low MOI (0.1) for persistently infected CT cells and co-infection scenarios, but only at certain time points. This discrepancy in results could be due to the interactions of MSV with each other (as previous studies have used only one MSV instead of a mixture of three MSVs). To be able to verify this possibility, it would be necessary to separate the three viruses. Theoretically, it should be possible to separate YicV from the two negeviruses due to their size difference (e.g. by gradient centrifugation). However, as DaesV and DeziV are very similar in size and have other similar molecular properties, the separation of these two viruses is difficult, especially since the classical plaque isolation method has been unsuccessful. In addition, several other factors that differ between our study and previous studies, such as the virus strains (MSVs and arboviruses), mosquito-derived cells and experimental set-ups (e.g. acute vs persistent infection), can also result in differences in virus-virus interactions. In previously published studies, the experiments were performed in Aedine-derived cells, instead of *Culex*-derived cells as used in our study. Until the present study, the limited research that has been performed in *Culex* spp. and derived cells focused only on the interaction of MSVs and arboviruses belonging to the same virus family [36–41]. As the mechanism of MSV inhibition on arboviruses is not known for most viruses, differences between* Aedes* and* Culex* could be possible. Furthermore, although we used arboviruses from the same families as the above-mentioned studies, the arbovirus strains we used differ from those in the previous studies. We focused on arboviruses that are relevant for* Culex* spp. (e.g. USUV, BUNV, SFV). The observed difference in the effects on arbovirus infection regarding persistently versus acutely infected cells could be (at least partly) due to changes in the relative amount of the viruses to each other (specifically DeziV/DaesV). Indeed, the DeziV/DaesV ratio changed from > 1 in acute infection to < 1 in persistently infected cells. However, this is only the case for CT cells and cannot explain the difference in Hsu cells, as in these cells both acute and persistently infected cells had a DeziV/Daes ratio < 1. Associated with this, a decrease in the relative virus concentration (specifically for DeziV) in acute versus persistently infected cells as well as between earlier passages of persistently infected CT cells (e.g. passage 4 or 5) and late passages (e.g. passage 17) was observed. For these experiments, various passages of the persistently infected CT cells were used, mostly from later passages (e.g. 12–20). Whether the lower amount of DeziV explains the observed differences regarding the interaction with arboviruses of acute or persistently infected cells cannot be concluded with certainty, as the decreased viral load of DeziV was only observed in CT cells and not Hsu cells.

Although CT and Hsu cells are both derived from* Culex* spp, they differ in their susceptibility to various arboviruses such as, for example, USUV (data not shown) and SFV. This difference has previously been reported for SFV [29] and again highlighted by the strong difference in luciferase activity in SFV-infected CT cells versus SFV-infected Hsu cells.

Viruses can be targeted by two RNAi pathways in mosquitoes, the siRNA and the piRNA pathway. All three viruses in this study only induced a siRNA response, and no typical ping-pong amplification-based piRNA response was observed. This is not surprising as all reported MSV-specific ping-pong-produced piRNAs are derived from negative-sense RNA viruses, like PCLV and MERDV [6, 7]. In the case of positive sense RNA or dsRNA MSVs (e.g. MeSV and NeVs), no production of ping-pong-produced piRNAs has been reported or reported only exclusively in cells, lacking the siRNA pathway [3, 6, 7, 42]. Interestingly, MeSV-specific sRNAs produced in aphids also showed a strong production of 22-nt siRNAs (size of siRNAs in aphids), mapping along the whole genome and antigenome, similar to our results in infected mosquito cells [43]. Together, these results suggest that dsRNA molecules (possibly replicate intermediates) are produced during YicV, DaesV and DeziV infection in mosquito-derived cells that are recognized and cleaved by Dicer-2. In the case of YicV infection in CT cells, the lack of YicV-specific siRNA production suggests that either no dsRNA molecules are produced, their amount is too low (for the used next-generation sequencing approach) or they are hidden from Dicer-2 recognition and cleavage.

The over-representation of DeziV- and DaesV-specific siRNAs from the terminal regions, especially the 5′ end region in persistently infected CT cells, which was not observed during acute infections, begs the question of whether targeting only this region is sufficient to control the infection to a non-pathogenic level, with still sufficient viral replication. Another possibility could be the alteration of the accessibility of the virus genomes during the course of an infection, such as through selective transcription from part of the genome. If these findings can be broadened to other MSVs or are NeV-specific is not known. However, several insect-specific flaviviruses (e.g. Cell-fusing agent virus [CFAV] and Calbertado virus) show an over-representation of the 3′-untranslated region in their produced siRNAs, independently of the infection status [17, 44, 45]. These produced siRNAs can potentially interfere with arboviruses if the targeted region is genetically conserved in both viruses, and future studies investigating the effect of vsiRNA produced upon insect-specific virus (ISV) infection might shed light on the mechanisms of ISV interference with arboviruses.

## Conclusions

Taken together, the discovery of a YicV variant and two NeVs (DeziV and DaesV), belonging to distinct clades, in a small (three mosquitoes) pool of *Cq. richiardii* mosquitoes in Germany expand their occurrence from previously reported countries worldwide to Germany. Therefore, these viruses support the wide geographical distribution and broad species host range of members of the *Mesoniviridae* and negeviruses. Production of vsiRNAs in *Aedes*- and *Culex*-derived cells shows the ability of these viruses to successfully induce the RNAi response upon infection. Infection of these MSVs in *Culex*-derived cells either has an inhibitory effect to various degrees or has no effect at all on co-infecting arboviruses. This study widens our understanding of the complex multipartite interaction of MSVs, mosquitoes and arboviruses.

### Supplementary Information

**Additional file 1:** **Table S1.** List of primers used 5′ to 3′.

**Additional file 2:** **Figure S1.** Generation of BUNV-NLuc.

**Additional file 3:** **Table S2.** YicV, DeziV and DaesV relative copy numbers (in 1.5 µg total RNA) in infected CT and Aag2 cells (72 hpi) from two independent experiments.

**Additional file 4:** **Table S3.** YicV, DeziV and DaesV relative copy numbers in persistently infected CT and Hsu cells or in the virus working stock.

**Additional file 5:** **Figure S2.** Effect of YicV/DeziV/DaesV infection status on Semliki Forest virus (SFV) and Bunyamwera orthobunya virus (BUNV) infections in *Culex quinquefasciatus-*derived (Hsu) cells.

**Additional file 6:** **Table S4.** YicV, DeziV and DaesV copy numbers (in 1.5 µg total RNA) in persistently infected CT and acutely infected CT cells (MOI 0.1, 24 hpi).

**Additional file 7.** **Figure S3.** Production of DaesV and DeziV-specific small RNAs in YicV/DeziV/DaesV acutely infected Aag2 or CT cells.

**Additional file 8:** **Figure S4.** Production of DaesV and DeziV-specific small RNAs in YicV/DeziV/DaesV persistently infected CT cells after beta-elemination treatment.

**Additional file 9**: **Table S5.** Summary Data on sRNA sequencing of DeziV, DaesV and YicV in Aag2 and CT cells. Roman numerals represent technical replicates.

**Additional file 10:** **Figure S5.** Mapping of DeziV and DaesV produced sRNAs (18–24 nts) on the corresponding other viral genome (DaesV sRNA mapped on DeziV genome and vice versa).

**Additional file 11:** **Figure S6.** Characterization of 26–30 nt long DaesV, DeziV and YiCV-specific small RNAs in acutely infected Aag2 cells.

**Additional file 12:** **Figure S7.** Characterization of 26–30 nt long DaesV and DeziV -specific small RNAs in acutely or persistently infected CT cells.

## Data Availability

The nucleotide sequences of the identified viruses are available in the NCBI Genbank: OP576001 (YicV8345), OP576002 (DaesV8345) and OP576003 (DeziV8345). Small RNA sequencing data and fastq files are available in the NCBI Sequence Read Archive under BioProject ID PRJNA885760. The luciferase, Sanger sequencing, complete gel pictures and RT-PCR data will be made available upon request.

## References

[1] Carvalho VL, Long MT (2021). Insect-specific viruses: an overview and their relationship to arboviruses of concern to humans and animals. Virology.

[2] Carvalho VL, Long MT. Perspectives on new vaccines against arboviruses using insect-specific viruses as platforms. Vaccines. 2021;9(3):263. 10.3390/vaccines9030263 PMC7999276 33809576

[3] Franzke K, Leggewie M, Sreenu VB, Jansen S, Heitmann A, Welch SR (2018). Detection, infection dynamics and small RNA response against Culex Y virus in mosquito-derived cells. J Gen Virol.

[4] Fredericks AC, Russell TA, Wallace LE, Davidson AD, Fernandez-Sesma A, Maringer K (2019). Aedes aegypti (Aag2)-derived clonal mosquito cell lines reveal the effects of pre-existing persistent infection with the insect-specific bunyavirus Phasi Charoen-like virus on arbovirus replication. PLoS Negl Trop Dis.

[5] Olmo RP, Todjro YMH, Aguiar ERGR, de Almeida JPP, Armache JN, de Faria IJS, et al. Insect-specific viruses regulate vector competence in* Aedes aegypti* mosquitoes via expression of histone H4. bioRxiv. 2021. 10.1101/2021.06.05.447047.

[6] Ruckert C, Prasad AN, Garcia-Luna SM, Robison A, Grubaugh ND, Weger-Lucarelli J (2019). Small RNA responses of Culex mosquitoes and cell lines during acute and persistent virus infection. Insect Biochem Mol Biol.

[7] Goertz GP, Miesen P, Overheul GJ, van Rij RP, van Oers MM, Pijlman GP. Mosquito small RNA responses to West Nile and insect-specific virus infections in* Aedes* and* Culex* mosquito cells. Viruses. 2019;11:271. 10.3390/v11030271 PMC6466260 30889941

[8] Hermanns K, Marklewitz M, Zirkel F, Overheul GJ, Page RA, Loaiza JR (2020). Agua Salud alphavirus defines a novel lineage of insect-specific alphaviruses discovered in the New World. J Gen Virol.

[9] Nasar F, Palacios G, Gorchakov RV, Guzman H, Da Rosa AP, Savji N (2012). Eilat virus, a unique alphavirus with host range restricted to insects by RNA replication. Proc Natl Acad Sci USA.

[10] Blair CD (2011). Mosquito RNAi is the major innate immune pathway controlling arbovirus infection and transmission. Future Microbiol.

[11] Miesen P, Joosten J, van Rij RP (2016). PIWIs go viral: arbovirus-derived piRNAs in vector mosquitoes. PLoS Pathog.

[12] Aguiar ER, Olmo RP, Paro S, Ferreira FV, de Faria IJ, Todjro YM (2015). Sequence-independent characterization of viruses based on the pattern of viral small RNAs produced by the host. Nucleic Acids Res.

[13] Scott JC, Brackney DE, Campbell CL, Bondu-Hawkins V, Hjelle B, Ebel GD (2010). Comparison of dengue virus type 2-specific small RNAs from RNA interference-competent and -incompetent mosquito cells. PLoS Negl Trop Dis.

[14] Liu J, Swevers L, Kolliopoulou A, Smagghe G (2019). Arboviruses and the challenge to establish systemic and persistent infections in competent mosquito vectors: the interaction with the RNAi mechanism. Front Physiol.

[15] Agboli E, Zahouli JBZ, Badolo A, Jost H. Mosquito-associated viruses and their related mosquitoes in West Africa. Viruses. 2021;13(5):891. 10.3390/v13050891 PMC8151702 34065928

[16] Martinet JP, Ferte H, Failloux AB, Schaffner F, Depaquit J. Mosquitoes of North-Western Europe as potential vectors of arboviruses: a review. Viruses. 2019;11(11):1059. 10.3390/v11111059 PMC6893686 31739553

[17] Varjak M, Maringer K, Watson M, Sreenu VB, Fredericks AC, Pondeville E, et al.* Aedes aegypti* Piwi4 is a noncanonical PIWI protein involved in antiviral responses. mSphere. 2017;2(3). 10.1128/mSphere.00144-17 PMC5415634 28497119

[18] Buchholz UJ, Finke S, Conzelmann KK (1999). Generation of bovine respiratory syncytial virus (BRSV) from cDNA: BRSV NS2 is not essential for virus replication in tissue culture, and the human RSV leader region acts as a functional BRSV genome promoter. J Virol.

[19] Börstler J. Arboviruses in Germany: geographical distribution and the interaction between mosquitoes and vertebrates. PhD thesis, University of Hamburg; 2010.

[20] Kopp A, Hubner A, Zirkel F, Hobelsberger D, Estrada A, Jordan I, et al. Detection of two highly diverse peribunyaviruses in mosquitoes from Palenque, Mexico. Viruses. 2019;11(9):832. 10.3390/v11090832 PMC6783978 31500304

[21] Cadar D, Luhken R, van der Jeugd H, Garigliany M, Ziegler U, Keller M, et al. Widespread activity of multiple lineages of Usutu virus, western Europe, 2016. Euro Surveillance. 2017;22(4). 10.2807/1560-7917.ES.2017.22.4.30452 PMC5388094 28181903

[22] Scherer C, Knowles J, Sreenu VB, Fredericks AC, Fuss J, Maringer K, et al. An* Aedes aegypti*-derived Ago2 knockout cell line to investigate arbovirus infections. Viruses. 2021;13(6):1066. 10.3390/v13061066 PMC8227176 34205194

[23] Dietrich I, Shi X, McFarlane M, Watson M, Blomstrom AL, Skelton JK (2017). The antiviral RNAi response in vector and non-vector cells against orthobunyaviruses. PLoS Negl Trop Dis.

[24] Tomazatos A, von Possel R, Pekarek N, Holm T, Rieger T, Baum H (2021). Discovery and genetic characterization of a novel orthonairovirus in Ixodes ricinus ticks from Danube Delta. Infect Genet Evol.

[25] Grabherr MG, Haas BJ, Yassour M, Levin JZ, Thompson DA, Amit I (2011). Full-length transcriptome assembly from RNA-Seq data without a reference genome. Nat Biotechnol.

[26] Fallmann J, Videm P, Bagnacani A, Batut B, Doyle MA, Klingstrom T, et al. The RNA workbench 2.0: next generation RNA data analysis. Nucleic Acids Res. 2019;47(W1):W511–W515. 10.1093/nar/gkz353 PMC6602469 31073612

[27] Antoniewski C (2014). Computing siRNA and piRNA overlap signatures. Methods Mol Biol.

[28] Altinli M, Leggewie M, Schulze J, Gyanwali R, Badusche M, Sreenu VB, et al. Antiviral RNAi Response in* Culex quinquefasciatus*-derived HSU cells. Viruses. 2023;15(2):436. 10.3390/v15020436 PMC9968050 36851650

[29] Varjak M, Leggewie M, Schnettler E (2018). The antiviral piRNA response in mosquitoes?. J Gen Virol.

[30] Kallies R, Kopp A, Zirkel F, Estrada A, Gillespie TR, Drosten C (2014). Genetic characterization of goutanap virus, a novel virus related to negeviruses, cileviruses and higreviruses. Viruses.

[31] Vasilakis N, Guzman H, Firth C, Forrester NL, Widen SG, Wood TG (2014). Mesoniviruses are mosquito-specific viruses with extensive geographic distribution and host range. Virol J.

[32] Nunes MRT, Contreras-Gutierrez MA, Guzman H, Martins LC, Barbirato MF, Savit C (2017). Genetic characterization, molecular epidemiology, and phylogenetic relationships of insect-specific viruses in the taxon Negevirus. Virology.

[33] Ferreira DD, Cook S, Lopes A, de Matos AP, Esteves A, Abecasis A (2013). Characterization of an insect-specific flavivirus (OCFVPT) co-isolated from *Ochlerotatus caspius* collected in southern Portugal along with a putative new Negev-like virus. Virus Genes.

[34] Patterson EI, Kautz TF, Contreras-Gutierrez MA, Guzman H, Tesh RB, Hughes GL (2021). Negeviruses reduce replication of alphaviruses during coinfection. J Virol.

[35] Ye G, Wang Y, Liu X, Dong Q, Cai Q, Yuan Z (2020). Transmission competence of a new mesonivirus, Yichang virus, in mosquitoes and its interference with representative flaviviruses. PLoS Negl Trop Dis.

[36] Newman CM, Cerutti F, Anderson TK, Hamer GL, Walker ED, Kitron UD (2011). Culex flavivirus and West Nile virus mosquito coinfection and positive ecological association in Chicago, United States. Vector Borne Zoonotic Dis.

[37] Crockett RK, Burkhalter K, Mead D, Kelly R, Brown J, Varnado W (2012). Culex flavivirus and West Nile virus in Culex quinquefasciatus populations in the southeastern United States. J Med Entomol.

[38] Bolling BG, Olea-Popelka FJ, Eisen L, Moore CG, Blair CD (2012). Transmission dynamics of an insect-specific flavivirus in a naturally infected Culex pipiens laboratory colony and effects of co-infection on vector competence for West Nile virus. Virology.

[39] Hall-Mendelin S, McLean BJ, Bielefeldt-Ohmann H, Hobson-Peters J, Hall RA, van den Hurk AF (2016). The insect-specific Palm Creek virus modulates West Nile virus infection in and transmission by Australian mosquitoes. Parasit Vectors.

[40] Goenaga S, Kenney JL, Duggal NK, Delorey M, Ebel GD, Zhang B (2015). Potential for co-infection of a mosquito-specific Flavivirus, Nhumirim virus, to block west Nile virus transmission in mosquitoes. Viruses.

[41] Kent RJ, Crabtree MB, Miller BR (2010). Transmission of West Nile virus by Culex quinquefasciatus say infected with Culex Flavivirus Izabal. PLoS Negl Trop Dis.

[42] Altinli M, Leggewie M, Badusche M, Gyanwali R, Scherer C, Schulze J, et al. Antiviral RNAi response against the insect-specific Agua Salud alphavirus. mSphere. 2022;7(1):e0100321. 10.1128/msphere.01003-21 PMC8849343 35171691

[43] Chang T, Guo M, Zhang W, Niu J, Wang JJ. First report of a mesonivirus and its derived small RNAs in an aphid species *Aphis citricidus* (Hemiptera: Aphididae), implying viral infection activity. J Insect Sci. 2020;20(2). 10.1093/jisesa/ieaa022 PMC7153580 32282036

[44] Schnettler E, Donald CL, Human S, Watson M, Siu RWC, McFarlane M (2013). Knockdown of piRNA pathway proteins results in enhanced Semliki Forest virus production in mosquito cells. J Gen Virol.

[45] Besson B, Overheul GJ, Wolfinger MT, Junglen S, Van Rij RP. Pan-flavivirus analysis reveals that the insect-specific Kamiti River virus produces a new subgenomic RNA and high amounts of 3´UTR-derived siRNAs. bioRxiv. 2022. 10.1101/2022.08.18.504478.

