# Supplementary material for: Interaction of Mesonivirus and Negevirus with arboviruses and the RNAi response in Culex tarsalis-derived cells
Source: Parasit Vectors. 2023 Oct 13;16:361. doi: 10.1186/s13071-023-05985-w (PMC10576325; doi:10.1186/s13071-023-05985-w)
Supplement: Supplementary file 1 — Additional file 1: Table S1. List of primers used 5′ to 3′. [file 13071_2023_5985_MOESM1_ESM.docx]

**Table S1** List of primers used. 5´to 3´.

| **Name** | **Sequence (5´-3´)** |
| --- | --- |
| Dezi-FW | ACGCCCCAAATGGAATCTGG |
| Dezi-RV | TCGTTCACGAATAGCGGCAG |
| DaesV-FW | TGACCGCAACAAGGGAGAAAC |
| DaesV-RV | TCAGAAGGGTGGACACCATGAC |
| YicV-FW | ATTGCCTCCACCAAGAGAGC |
| YicV-RV | ATGGCGTCTAGAGTCTCGGT |
| MeSV-FW | TGGHGATKCRGAATTCATGCG |
| MeSV-RV | ATCCCAACCRCCRTATTGTGC |
| Dezi-qFW | GGTTCTGCTGCCACGTATTT |
| Dezi-qRV | ATTTTTCGAACCTCGGTGTG |
| DaesV-qFW | ATGCGTCTTCACTTTGTCGG |
| DaesV-qRV | CGGTCAATAACGTCCACCAA |
| YicV-qFW | GCAGAAACTTACGGCTATGCAG |
| YicV-qRV | GGAGAGCTGATGTTCAAGCG |
