# Supplementary material for: Interaction of Mesonivirus and Negevirus with arboviruses and the RNAi response in Culex tarsalis-derived cells
Source: Parasit Vectors. 2023 Oct 13;16:361. doi: 10.1186/s13071-023-05985-w (PMC10576325; doi:10.1186/s13071-023-05985-w)
Supplement: Supplementary file 2 — Additional file 2: Figure S1. Generation of BUNV-NLuc. [file 13071_2023_5985_MOESM2_ESM.docx]

**
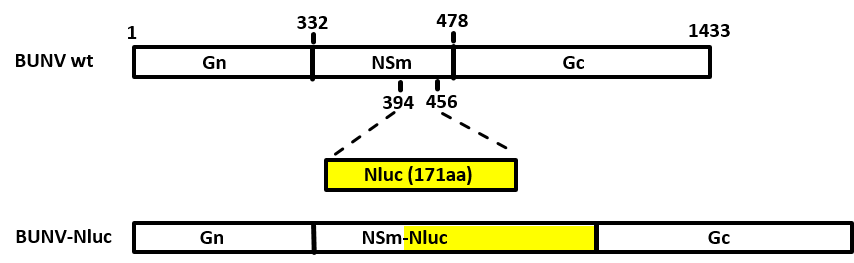
**

**Figure S1: Generation of BUNV-NLuc**

Outline of the M-segment of BUNV wt and BUNV-NLuc. In BUNV-Nluc, the coding region of the NSm cytoplasmic tail (residues 395 to 455) was replaced by Nano luciferase (NLuc); resulting in NSm-NLuc chimeric protein cleaved between Gn and Gc proteins. Adapted from (Dietrich, Shi, et al. 2017; <https://doi.org/10.1371/journal.pntd.0005272>).
