# Supplementary material for: Interaction of Mesonivirus and Negevirus with arboviruses and the RNAi response in Culex tarsalis-derived cells
Source: Parasit Vectors. 2023 Oct 13;16:361. doi: 10.1186/s13071-023-05985-w (PMC10576325; doi:10.1186/s13071-023-05985-w)
Supplement: Supplementary file 3 — Additional file 3: Table S2. YicV, DeziV and DaesV relative copy numbers (in 1.5 µg total RNA) in infected CT and Aag2 cells (72 hpi) from two independent experiments. [file 13071_2023_5985_MOESM3_ESM.docx]

**Table S2** YicV, DeziV and DaesV relative copy numbers (in 1,5 µg total RNA) in infected CT and Aag2 cells (72hpi) from two independent experiments. NA, not applicable (due to the sensitivity of the qPCR, according to the standard curve, no reliable quantification was possible).

|  | **CT cells MOI1** | | | **Aag2 cells MOI1** | | | **Hsu cells MOI1** | | |
| --- | --- | --- | --- | --- | --- | --- | --- | --- | --- |
|  | **YicV** | **DeziV** | **DaesV** | **YicV** | **DeziV** | **DaesV** | **YicV** | **DeziV** | **DaesV** |
| **relative copy numbers (n=2/3)** | 9,4E+03  NA | 2,1E+08  7,4E+07 | 1,1E+08  3,3E+07 | 6,4E+06  2,9E+08 | 1,7E+07  4,1E+08 | 4,6E+07  1,7E+08 | NA  9,0E+2  4,7E+2 | 1,5E+4  3,6E+4  5,3E+3 | 3,9E+6  4,7E+6  1,3E+7 |
| **Ratio**  **(DeziV/ DaesV)** |  | 1,9  2,2 |  |  | 0,4  2,4 |  |  | 0,004  0,0077  0,0004 |  |
