# Supplementary material for: Interaction of Mesonivirus and Negevirus with arboviruses and the RNAi response in Culex tarsalis-derived cells
Source: Parasit Vectors. 2023 Oct 13;16:361. doi: 10.1186/s13071-023-05985-w (PMC10576325; doi:10.1186/s13071-023-05985-w)
Supplement: Supplementary file 4 — Additional file 4: Table S3. YicV, DeziV and DaesV relative copy numbers in persistently infected CT and Hsu cells or in the virus working stock. [file 13071_2023_5985_MOESM4_ESM.docx]

Table S3 YicV, DeziV, and DaesV relative copy numbers in persistently infected CT and Hsu cells or in the virus working stock. p represents the passage number of the persistently infected cells, after the initial infection. NA, not applicable (due to the sensitivity of the qPCR, according to the standard curve, no reliable quantification was possible).

|  | **Relative copy numbers**  **(in 1,5 µg total RNA)** | | |  |
| --- | --- | --- | --- | --- |
|  | **YicV** | **DeziV** | **DaesV** | **Ratio (DeziV/DaesV)** |
| **CT p3** | 5,3x10^2^ | 4,9 x10^7^ | 3,6 x10^7^ | 1,37 |
| **CT p4** | 4,9 x10^2^ | 5,2 x10^7^ | 3,7 x10^7^ | 1,41 |
| **CT p16** | 6,9 x10^2^ | 2,9 x10^6^ | 7,7 x10^7^ | 0,04 |
| **CT p20** | NA | 2,2 x10^5^ | 7,1 x10^6^ | 0,03 |
|  |  |  |  |  |
| **Hsu p4** | 5,1 x10^2^ | 6,9 x10^5^ | 5,0 x10^6^ | 0,14 |
| **Hsu p5** | 1,4 x10^3^ | 7,7 x10^4^ | 2,5 x10^6^ | 0,03 |
| **Hsu p17** | NA | 5,1 x10^4^ | 2,7 x10^6^ | 0,02 |
|  | **Relative copy numbers/ml** | | |  |
| **Virus working stock** | 6,3 x10^9^ | 1,0 x10^12^ | 3,1 x10^11^ | 3,25 |
