# Supplementary material for: Interaction of Mesonivirus and Negevirus with arboviruses and the RNAi response in Culex tarsalis-derived cells
Source: Parasit Vectors. 2023 Oct 13;16:361. doi: 10.1186/s13071-023-05985-w (PMC10576325; doi:10.1186/s13071-023-05985-w)
Supplement: Supplementary file 5 — Additional file 5: Figure S2. Effect of YicV/DeziV/DaesV infection status on Semliki Forest virus (SFV) and Bunyamwera orthobunya virus (BUNV) infections in Culex quinquefasciatus-derived (Hsu) cells. [file 13071_2023_5985_MOESM5_ESM.docx]

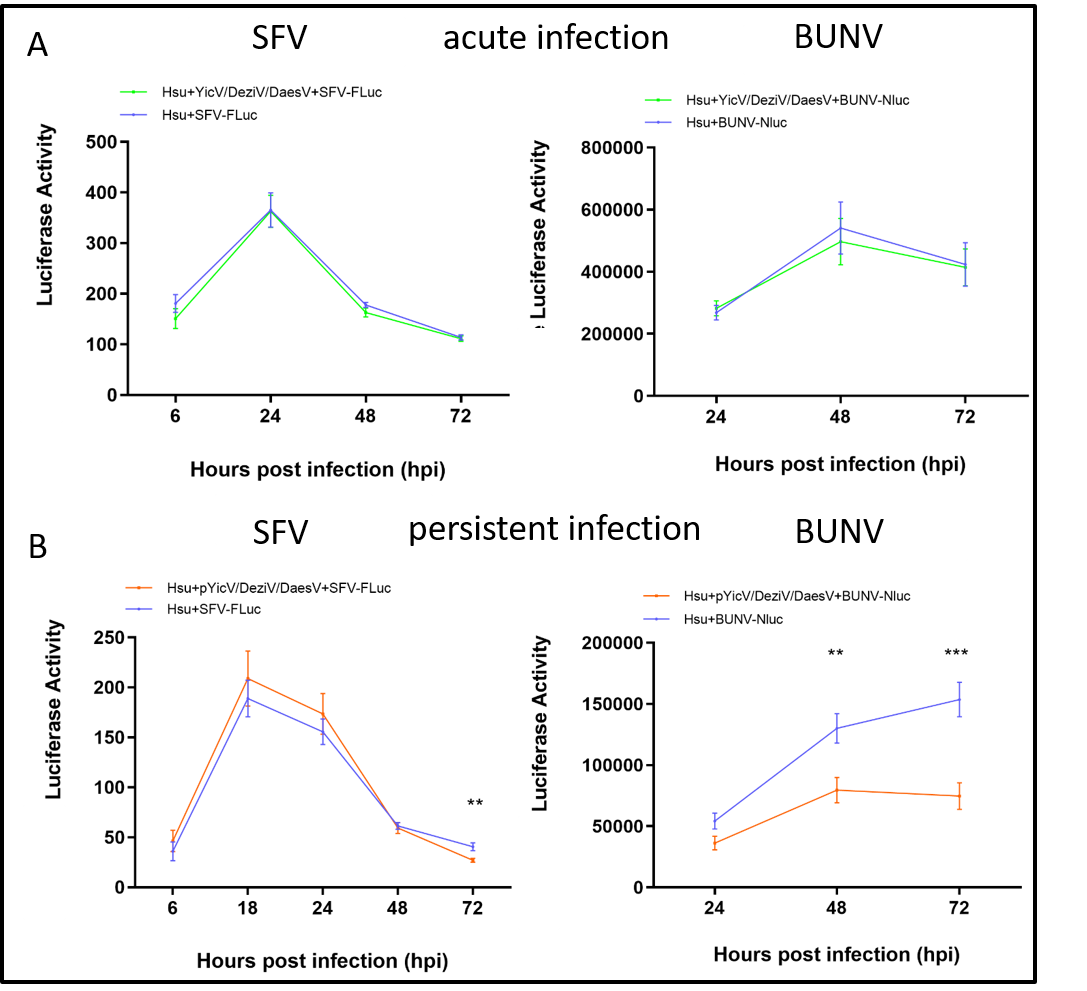


**Fig. S2** Effect of YicV/DeziV/DaesV infection status on Semliki Forest virus (SFV) and Bunyamwera orthobunya virus (BUNV) infections in *Culex quinquefasciatus-derived* (Hsu) cells**.**

Culex quinquefasciatus-derived Hsu cells were single infected with SFV-FLuc (Hsu+SFV-FLuc; MOI 10) or BUNV-NLuc (Hsu+BUNV-NLuc; MOI 0.1) and compared to cells acutely co-infected with YicV/DeziV/DaesV and SFV-FLuc (Hsu+ YicV/DeziV/DaesV +SFV-FLuc; MOI 10) or BUNV-NLuc (Hsu+ YicV/DeziV/DaesV +BUNV-NLuc; MOI 0.1) (A) or compared to SFV-FLuc (Hsu+pYicV/DeziV/DaesV +SFV-FLuc; MOI 10) or BUNV-NLuc (Hsu+p YicV/DeziV/DaesV +BUNV-NLuc; MOI 0.1) infection in YicV/DeziV/DaesV persistently infected cells (B). Cells were lysed at indicated time points and Luciferase activity was measured. Results of three independent experiments (using different passages of persistent infected Hsu cells, from 10^th^ to 13^th^ passage) performed in technical triplicate are presented. Mean values with SEM are shown. Significance was tested with unpaired t-test (SFV-FLuc=*p<0.05: **p=0.0071; BUNV-NLuc=*p<0.05: **p=0.0058, ***p=0.0004).
