# Supplementary material for: Interaction of Mesonivirus and Negevirus with arboviruses and the RNAi response in Culex tarsalis-derived cells
Source: Parasit Vectors. 2023 Oct 13;16:361. doi: 10.1186/s13071-023-05985-w (PMC10576325; doi:10.1186/s13071-023-05985-w)
Supplement: Supplementary file 6 — Additional file 6: Table S4. YicV, DeziV and DaesV copy numbers (in 1.5 µg total RNA) in persistently infected CT and acutely infected CT cells (MOI 0.1, 24 hpi). [file 13071_2023_5985_MOESM6_ESM.docx]

**Table S4** YicV, DeziV and DaesV copy numbers (in 1,5 µg total RNA) in persistently infected CT and acutely infected CT cells (MOI 0.1, 24hpi). NA, not applicable (due to the sensitivity of the qPCR, according to the standard curve, no reliable quantification was possible).

|  | **CT persistent cells** | | | **CT MOI 0.1 (24hpi)** | | |
| --- | --- | --- | --- | --- | --- | --- |
|  | **YicV** | **DeziV** | **DaesV** | **YicV** | **DeziV** | **DaesV** |
| **Average copy numbers** | NA | 2,2 x10^5^ | 7,1 x10^6^ | NA | 1,1 x10^8^ | 8,1 x10^7^ |
| **Ratio (DeziV/DaesV)** |  | 0,03 |  |  | 1,37 |  |
