# Supplementary material for: Interaction of Mesonivirus and Negevirus with arboviruses and the RNAi response in Culex tarsalis-derived cells
Source: Parasit Vectors. 2023 Oct 13;16:361. doi: 10.1186/s13071-023-05985-w (PMC10576325; doi:10.1186/s13071-023-05985-w)
Supplement: Supplementary file 8 — Additional file 8: Figure S4. Production of DaesV and DeziV-specific small RNAs in YicV/DeziV/DaesV persistently infected CT cells after beta-elemination treatment. [file 13071_2023_5985_MOESM8_ESM.docx]

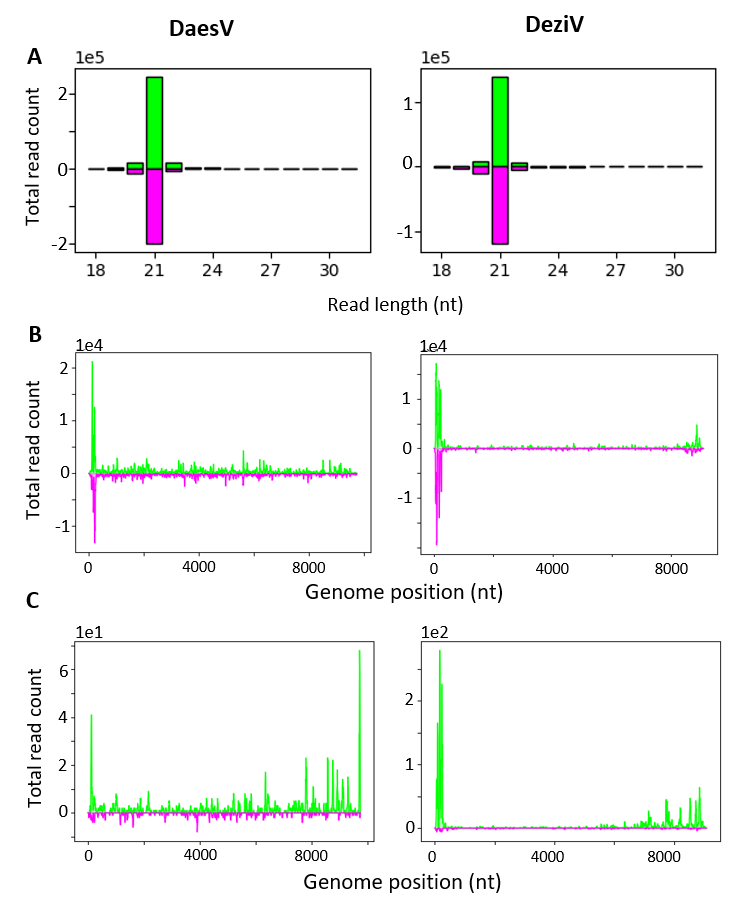


Fig. S4 Production of DaesV and DeziV-specific small RNAs in YicV/DeziV/DaesV persistently infected CT cells after beta-elemination treatment.

RNA of persistently infected CT cells were isolated, followed by β-elimination treatment or control (Fig. 2 and 3). (A) the absolute frequency of length of sRNAs from 18 – 31 nt that were mapped to the virus genome/ antigenome. (B) the distribution of 21 nt long sRNA to the indicated virus genome/ antigenome. (C) shows the distribution of 26 – 30 nt long sRNAs (piRNA-sized) to the virus genome/ antigenome. Positive values (green) represent sense, negative values (purple), antisense reads. Y-scale values give the read counts, with the scale values mentioned above the graph.
