# Supplementary material for: Interaction of Mesonivirus and Negevirus with arboviruses and the RNAi response in Culex tarsalis-derived cells
Source: Parasit Vectors. 2023 Oct 13;16:361. doi: 10.1186/s13071-023-05985-w (PMC10576325; doi:10.1186/s13071-023-05985-w)
Supplement: Supplementary file 9 — Additional file 9: Table S5. Summary Data on sRNA sequencing of DeziV, DaesV and YicV in Aag2 and CT cells. Roman numerals represent technical replicates. [file 13071_2023_5985_MOESM9_ESM.docx]

**Supplementary data, Agboli, Schulze et al.**

**Table S5** Summary Data on sRNA sequencing of DeziV, DaesV and YicV in Aag2 and CT cells. Roman numerals represent technical replicates. nd, not detectable.
