# Supplementary material for: Interaction of Mesonivirus and Negevirus with arboviruses and the RNAi response in Culex tarsalis-derived cells
Source: Parasit Vectors. 2023 Oct 13;16:361. doi: 10.1186/s13071-023-05985-w (PMC10576325; doi:10.1186/s13071-023-05985-w)
Supplement: Supplementary file 10 — Additional file 10: Figure S5. Mapping of DeziV and DaesV produced sRNAs (18–24 nts) on the corresponding other viral genome (DaesV sRNA mapped on DeziV genome and vice versa). [file 13071_2023_5985_MOESM10_ESM.docx]

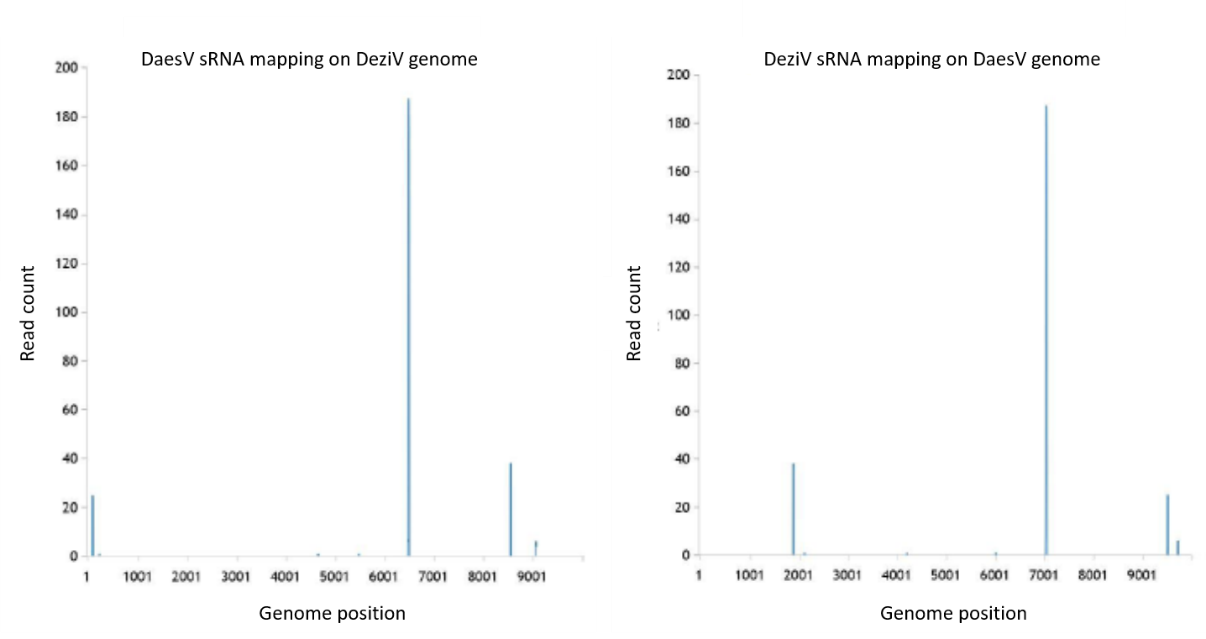


**Fig. S5** Mapping of DeziV and DaesV produced sRNAs (18-24 nts) on the corresponding other viral genome (DaesV sRNA mapped on DeziV genome and vice versa).
