# Supplementary material for: Interaction of Mesonivirus and Negevirus with arboviruses and the RNAi response in Culex tarsalis-derived cells
Source: Parasit Vectors. 2023 Oct 13;16:361. doi: 10.1186/s13071-023-05985-w (PMC10576325; doi:10.1186/s13071-023-05985-w)
Supplement: Supplementary file 11 — Additional file 11: Figure S6. Characterization of 26–30 nt long DaesV, DeziV and YiCV-specific small RNAs in acutely infected Aag2 cells. [file 13071_2023_5985_MOESM11_ESM.docx]

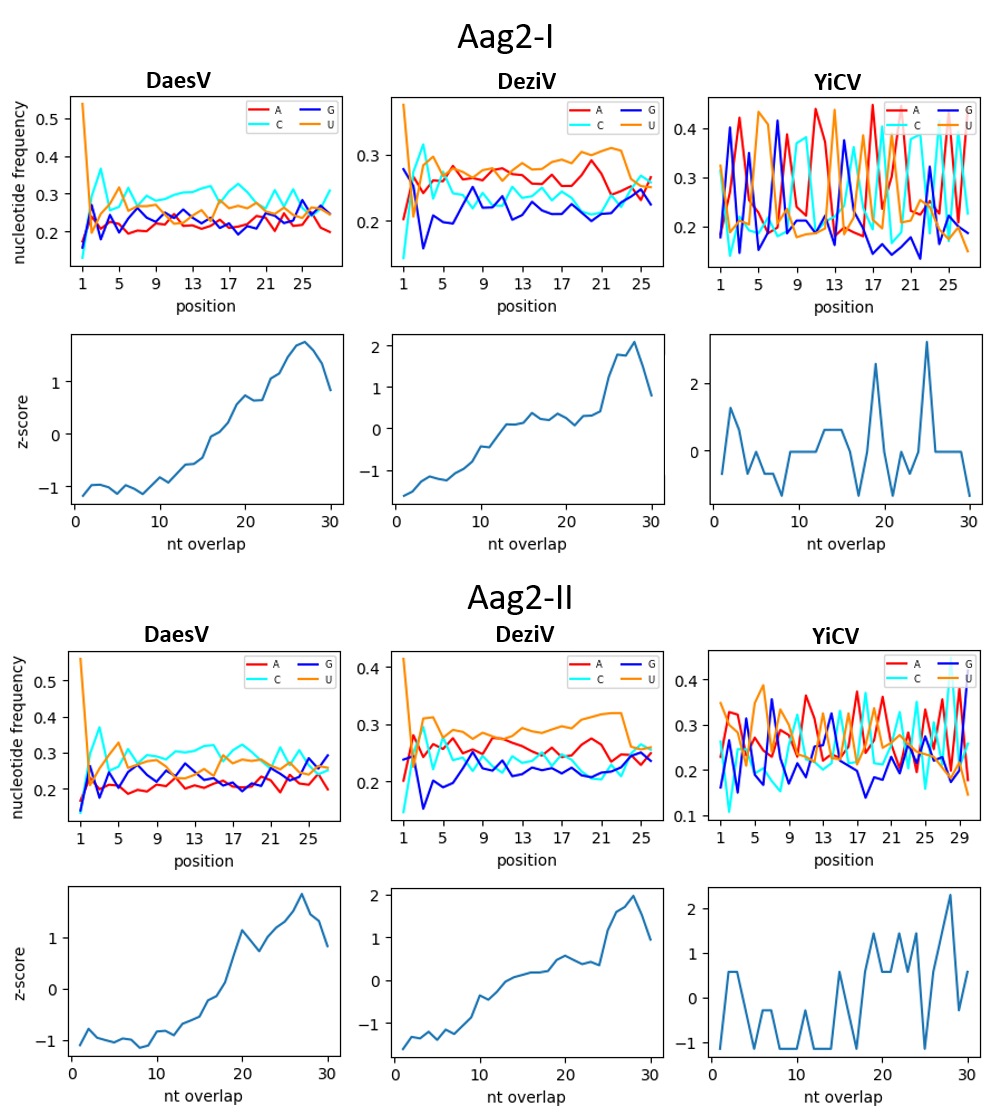


**Fig. S6** Characterization of 26-30 nt long DaesV, DeziV and YiCV-specific small RNAs in acutely infected Aag2 cells.

Aag2 cells were infected with the DaesV/DeziV/ YicV mix and total RNA was isolated at 24 h post infection. (A) Sequence logo plots showing the sequence bias in various positions. (B) Overlap frequencies of sense and antisense 26- to 30-nt-long DaesV/DeziV/ YicV -specific small RNAs. Results from two independent experiments (I and II).
