# Supplementary material for: Interaction of Mesonivirus and Negevirus with arboviruses and the RNAi response in Culex tarsalis-derived cells
Source: Parasit Vectors. 2023 Oct 13;16:361. doi: 10.1186/s13071-023-05985-w (PMC10576325; doi:10.1186/s13071-023-05985-w)
Supplement: Supplementary file 12 — Additional file 12: Figure S7. Characterization of 26–30 nt long DaesV and DeziV -specific small RNAs in acutely or persistently infected CT cells. [file 13071_2023_5985_MOESM12_ESM.docx]

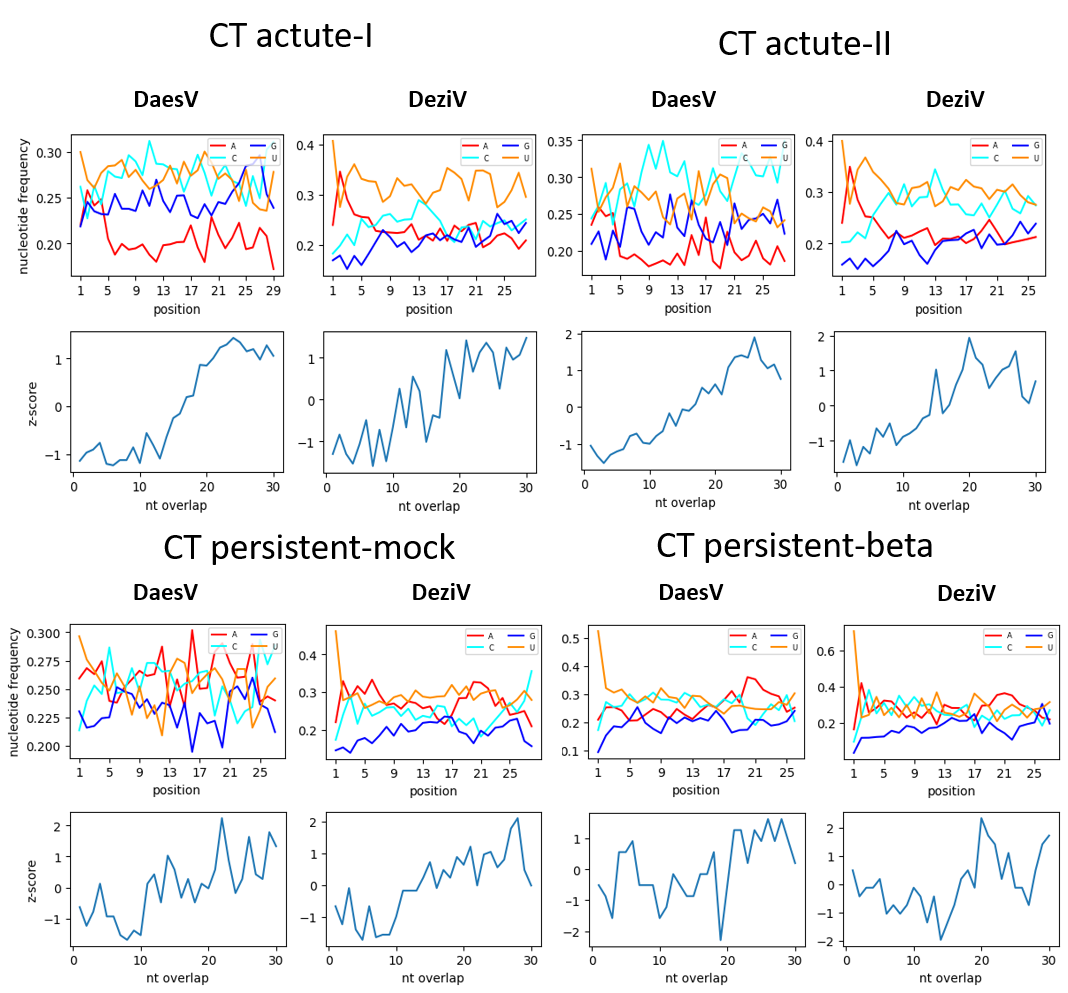


**Fig. S7** Characterization of 26-30 nt long DaesV and DeziV -specific small RNAs in acutely or persistently infected CT cells.

For acute infection, cell were infected with the DaesV/DeziV/ YicV mix and total RNA was isolated at 24 h post infection. RNA of persistently infected CT cells were isolated, followed by β-elimination treatment (CT-persistent beta) and control (CT persistent-mock). (A) Sequence logo plots showing the sequence bias in various positions. (B) Overlap frequencies of sense and antisense 26- to 30-nt-long DaesV and DeziV-specific small RNAs.
